# Assessing rates and predictors of cannabis-associated psychotic symptoms across observational, experimental and medical research

**DOI:** 10.1038/s44220-024-00261-x

**Published:** 2024-06-03

**Authors:** Tabea Schoeler, Jessie R. Baldwin, Ellen Martin, Wikus Barkhuizen, Jean-Baptiste Pingault

**Affiliations:** 1 Department of Computational Biology, University of Lausanne, Lausanne, Switzerland; 2 Department of Clinical, Educational and Health Psychology, Division of Psychology and Language Sciences, University College London, London, UK; 3 Social, Genetic and Developmental Psychiatry Centre, Institute of Psychiatry, Psychology and Neuroscience, King’s College London, London, UK

**Keywords:** Risk factors, Outcomes research

## Abstract

Cannabis, one of the most widely used psychoactive substances worldwide, can give rise to acute cannabis-associated psychotic symptoms (CAPS). While distinct study designs have been used to examine CAPS, an overarching synthesis of the existing findings has not yet been carried forward. To that end, we quantitatively pooled the evidence on rates and predictors of CAPS (*k* = 162 studies, *n* = 210,283 cannabis-exposed individuals) as studied in (1) observational research, (2) experimental tetrahydrocannabinol (THC) studies, and (3) medicinal cannabis research. We found that rates of CAPS varied substantially across the study designs, given the high rates reported by observational and experimental research (19% and 21%, respectively) but not medicinal cannabis studies (2%). CAPS was predicted by THC administration (for example, single dose, Cohen’s *d* = 0.7), mental health liabilities (for example, bipolar disorder, *d* = 0.8), dopamine activity (*d* = 0.4), younger age (*d* = −0.2), and female gender (*d* = −0.09). Neither candidate genes (for example, *COMT*, *AKT1*) nor other demographic variables (for example, education) predicted CAPS in meta-analytical models. The results reinforce the need to more closely monitor adverse cannabis-related outcomes in vulnerable individuals as these individuals may benefit most from harm-reduction efforts.

## Abstract (web summary)

The authors synthesize data from previous literature on observational, experimental and medicinal cannabis research to assess rates and predictors of cannabis-associated psychotic symptoms.

## Main

Cannabis, one of the most widely used psychoactive substances in the world,^1^ is commonly used as a recreational substance and is increasingly taken for medicinal purposes.^2,3^ As a recreational substance, cannabis use is particularly prevalent among young people^1^ who seek its rewarding acute effects such as relaxation, euphoria, or sociability.^4^ When used as a medicinal product, cannabis is typically prescribed to alleviate clinical symptoms in individuals with pre-existing health conditions (for example, epilepsy, multiple sclerosis, chronic pain, nausea.^5^)

Given the widespread use of cannabis, alongside the shifts toward legalization of cannabis for medicinal and recreational purposes, momentum is growing to scrutinize both the potential therapeutic and adverse effects of cannabis on health. From a public health perspective, of particular concern are the increasing rates of cannabis-associated emergency department presentations,^6^ the rising levels of THC (tetrahydrocannabinol, the main psychoactive ingredient in cannabis) in street cannabis,^7^ the adverse events associated with medicinal cannabis use,^8^ and the long-term health hazards associated with cannabis use.^9^ In this context, risk of psychosis as a major adverse health outcome related to cannabis use has been studied extensively, suggesting that early-onset and heavy cannabis use constitutes a contributory cause of psychosis.^10–12^

More recent research has started to examine the more acute cannabis-associated psychotic symptoms (CAPS) to understand better how individual vulnerabilities and the pharmacological properties of cannabis elicit adverse reactions in individuals exposed to cannabis. Indeed, transient psychosis-like symptoms, including hallucinations or paranoia during cannabis intoxication, are well documented.^5,13,14^ In more rare cases, recreational cannabis users experience severe forms of CAPS,^15^ requiring emergency medical treatment as a result of acute CAPS.^16^ In addition, acute psychosis following THC administration has been documented in medicinal cannabis trials and experimental studies,^17–19^ suggesting that CAPS can also occur in more-controlled environments.

While numerous studies have provided evidence on CAPS in humans, no research has yet synthesized and compared the findings obtained from different study designs and populations. More specifically, three distinct study types have focused on CAPS: (1) observational studies assessing the subjective experiences of cannabis intoxication in recreational cannabis users, (2) experimental challenge studies administering THC in healthy volunteers, and (3) medicinal cannabis studies documenting adverse events when testing medicinal cannabis products in individuals with pre-existing health conditions. As such, the availability of these three distinct lines of evidence provides a unique research opportunity as their findings can be synthesized, be inspected for convergence, and ultimately, contribute to more evidence-based harm-reduction initiatives.

In this work, we therefore aim to perform a quantitative synthesis of all existing evidence examining CAPS to advance our understanding concerning the rates and predictors of CAPS: First, it is currently unknown how common CAPS are among individuals exposed to cannabis. While rates of CAPS are reported by numerous studies, estimates vary substantially (for example, from <1% (ref. ^20^) to 70% (ref. ^21^)) and may differ depending on the assessed symptom profile (for example, cannabis-associated hallucinations versus cannabis-associated paranoia), the study design (for example, observational versus experimental research), and the population (for example, healthy volunteers versus medicinal cannabis users). Second, distinct study designs have scrutinized similar questions concerning the risks involved in CAPS. As such, comparisons of the results from one study design (for example, observational studies, assessing self-reported cannabis use in recreational users^22,23^) with another study design (for example, experimental studies administering varying doses of THC^24,25^) can be used to triangulate findings on a given risk factor of interest (for example, potency of cannabis). Finally, studies focusing on predictors of CAPS typically assess hypothesized risk factors in isolation. Pooling all existing evidence across different risk factors therefore provides a more complete picture of the relative magnitude of the individual risk factors involved in CAPS.

In summary, this work is set out to synthesize all of the available evidence on CAPS across three lines of research. In light of the increasingly liberal cannabis policies around the world, alongside the rising levels of THC in cannabis, such efforts are key to informing harm-reduction strategies and future research avenues for public health. Considering that individuals presenting with acute cannabis-induced psychosis are at high risk of converting to a psychotic disorder (for example, rates ranging between 18% (ref. ^26^) and 45% (ref. ^27^)), a deeper understanding of factors predicting CAPS would contribute to our understanding concerning risk of long-term psychosis in the context of cannabis use.

## Results

**Fig. 1:**
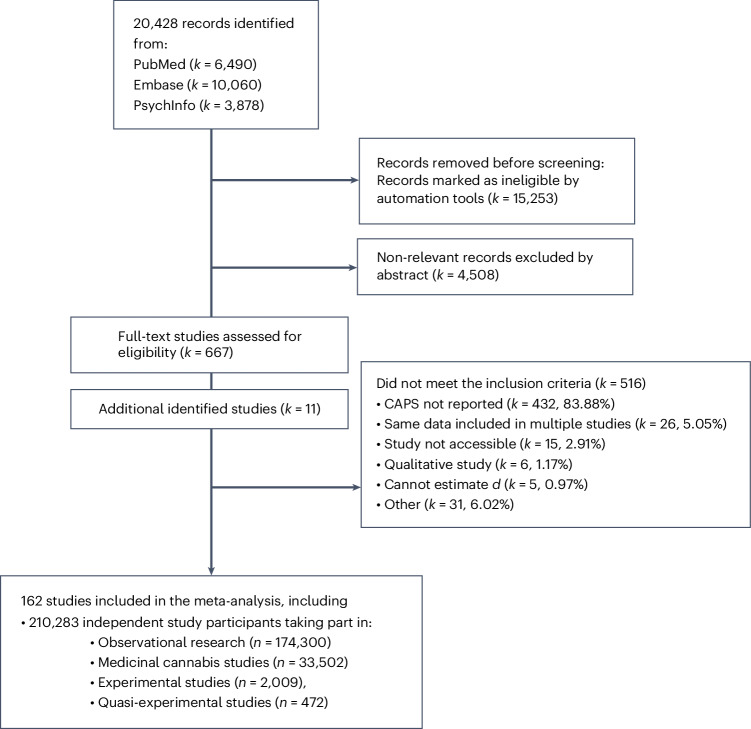
PRISMA flow chart. Flow chart as adapted from the PRISMA flow chart (http://www.prisma-statement.org/). Independent study participants are defined as the maximum number of participants available for an underlying study sample assessed in one or more of the included studies.

**Fig. 2:**
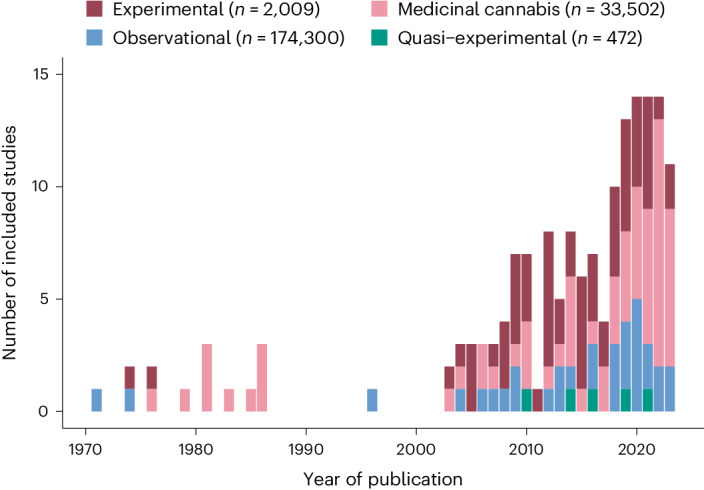
Number of included studies and study participants per study design according to year of publication. Number of included studies per year of publication and study design, including observational research assessing recreational cannabis users, experimental studies administering THC in healthy volunteers, and medicinal studies assessing adverse events in individuals taking cannabis products for medicinal use. Quasi-experimental research involved research testing the effects of THC administration in a naturalistic setting.^23,62^ *k*, number of studies; *n*, number of (non-overlapping) study participants.

Of 20,428 published studies identified by the systematic search, 162 were included in this work. The reasons for exclusion are detailed in the PRISMA (Preferred Reporting Items for Systematic Reviews and Meta-Analyses) flow diagram (Fig. 1; see Supplementary Fig. 1 for a breakdown of the number of independent participants included in the different analytical models). The PRISMA reporting checklist is included in the Supplementary Results. At the full-text screening stage, the majority of studies were excluded because they did not report data on CAPS (83.88% of all excluded studies). Figure 2 displays the number of published studies included (*k*) and the number of (non-overlapping) study participants (*n*) per study design, highlighting that out of all participants included in this meta-analysis (*n* = 201,283), most took part in observational research (*n* = 174,300; 82.89%), followed by studies assessing medicinal cannabis products (*n* = 33,502; 15.93%), experimental studies administering THC (*n* = 2,009; 0.96%), and quasi-experimental studies (*n* = 472; 0.22%). Screening of 10% of the studies at the full-text stage by an independent researcher (E.M.) did not identify missed studies.

### Rates of CAPS across the three study designs

A total of 99 studies published between 1971 and 2023 reported data on rates of CAPS and were included in the analysis, comprising 126,430 individuals from independent samples. Convergence of the data extracted by the two researchers (T.S. and W.B.) was high for the pooled rates on CAPS from observational studies (rate_DIFF_ = −0.01%, where rate_DIFF_ = rate_TS_ – rate_WB_), experimental studies (rate_DIFF_ = 0%), and medicinal cannabis studies (rate_DIFF_ = 0%). More specifically, we included data from 41 observational studies (*n* = 92,888 cannabis users), 19 experimental studies administering THC (*n* = 754), and 79 studies assessing efficacy and tolerability of medicinal cannabis products containing THC (*n* = 32,821). In medicinal trials, the most common conditions treated with THC were pain (*k* = 19 (23.75%)) and cancer (*k* = 16 (20%)) (see Supplementary Table 1 for an overview). The age distribution of the included participants was similar in observational studies (mean age = 24.47 years, ranging from 16.6 to 34.34 years) and experimental studies (mean age = 25.1 years, ranging from 22.47 to 27.3 years). Individuals taking part in medicinal trials were substantially older (mean age = 48.16 years, ranging from 8 to 74.5 years).

**Fig. 3:**
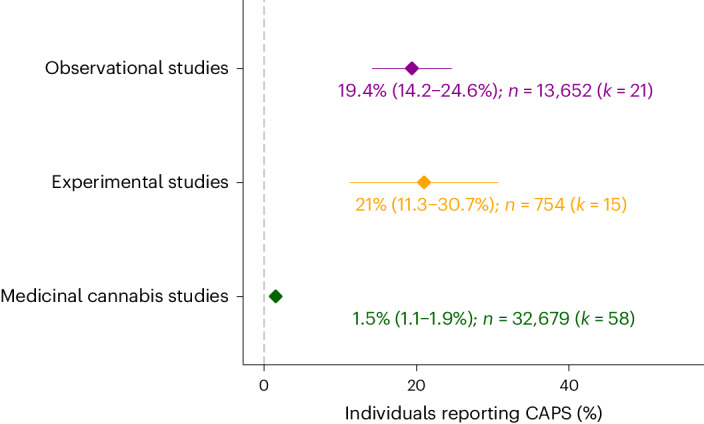
Rates of cannabis-associated psychotic symptoms across study designs. Pooled rates of CAPS across the three different study designs. Estimates on the *y* axis are the rates (in %, 95% confidence interval) obtained from models pooling together estimates on rates of CAPS (including psychosis-like symptoms, paranoia, hallucinations, and delusions) per study design.

As summarized in Fig. 3 and Supplementary Table 3, substantial rates of CAPS were reported by observational studies (19.4%, 95% confidence interval (CI): 14.2%, 24.6%) and THC-challenge studies (21%, 95% CI: 11.3%, 30.7%), but not medicinal cannabis studies (1.5%, 95% CI: 1.1%, 1.9%). The pooled rates estimated for different symptom profiles of CAPS (CAPS – paranoia, CAPS – hallucinations, CAPS – delusions) are displayed in Supplementary Fig. 2. All individual study estimates are listed in Supplementary Table 2.

**Fig. 4:**
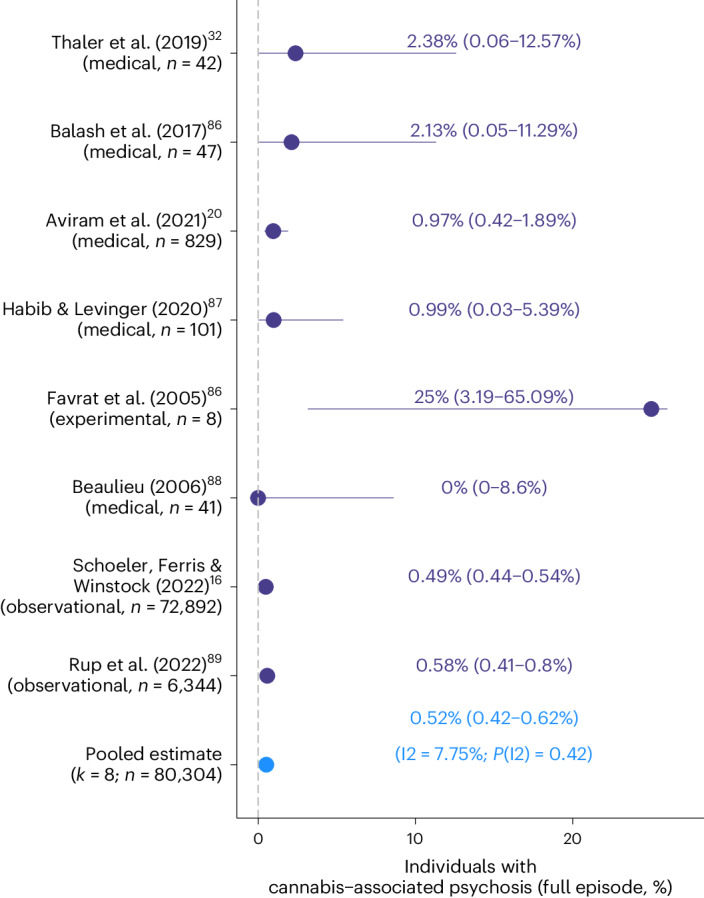
Rates of cannabis-associated psychosis (full episode). Studies reporting rates of cannabis-associated psychosis (full episode). Depicted in violet are the individual study estimates (in %, 95% confidence interval) of studies reporting rates of (full-blown) cannabis-associated psychotic episodes. Included are studies using medicinal cannabis, observational, or experimental samples. The pooled meta-analyzed estimate is colored in blue. The *I*^*2*^ statistic (scale of 0 to 100) indexes the level of heterogeneity across the estimates included in the meta-analysis.

Most models showed significant levels of heterogeneity (Supplementary Table 3), highlighting that rates of CAPS differed as a function of study-specific features. Risk of publication bias was indicated (*P*_Peters_ < 0.05) for one of the meta-analytical models combining all rates of CAPS (see funnel plots, Supplementary Fig. 2). Applying the trim-and-fill method slightly reduced the pooled rate of CAPS obtained from medicinal cannabis studies (rate_unadjusted_ = 1.53%; rate_adjusted_ = 1.18%). Finally, Fig. 4 summarizes rates of CAPS of a subset of studies where CAPS was defined as the occurrence of a full-blown cannabis-associated psychotic episode (as described in Table 1). When combined, the rate of CAPS (full episode) was 0.52% (0.42–0.62%) across the three study designs, highlighting that around one in 200 individuals experienced a severe episode of psychosis when exposed to cannabis/THC. Rates of CAPS (full episode) as reported by the individual studies showed high levels of consistency (*I*^*2*^ = 8%, *P(I*^*2*^) = 0.45; Fig. 4).

**Table 1:** Examples of measures assessing cannabis-associated psychotic symptoms

	Observational studies	THC-challenge studies	Medicinal cannabis studies
Design-specific definition of CAPS	Acute cannabis-associated psychotic experiences as retrospectively assessed using self-report measures in individuals using cannabis sampled from the general population	Degree of psychotic symptom change in response to THC, estimated from between-subject (placebo groups versus THC group) or within-subject (pre-THC versus post-THC assessment) comparisons in healthy participants	Adverse reactions involving psychosis resulting from the use of medicinal cannabis products containing THC
CAPS – psychosis(-like)	‘After taking cannabis, have you had strange, unpleasant experiences such as hearing voices or becoming convinced that someone is trying to harm you, or that you are being persecuted?’^78^	Changes in Brief Psychiatric Rating Scale following THC administration^23^	Occurrence of any psychotic symptom when taking medicinal cannabis products^79^
CAPS – hallucinations	‘How often have you experienced the following reactions to cannabis use, while high or under the influence of cannabis? Auditory or visual hallucinations’^80^	Changes in Positive and Negative Syndrome Scale (hallucinations) following THC administration^25^	Adverse events (hallucinations) when taking medicinal cannabis products^81^
CAPS – delusions	‘How often have you experienced the following reactions [delusional] to cannabis use, while under the influence of the substance?’^80^	Changes in Psychotomimetic States Inventory (delusion subscale) following THC administration^82^	Adverse events (delusions) related to treatment with medicinal cannabis^20^
CAPS – paranoia	‘How often have you felt suspicious while smoking cannabis?’^22^	Changes in Visual Analogue Scales (paranoia item) following THC administration^83^	Frequency of adverse events during medicinal cannabis trial (paranoia)^84^
CAPS – full episode	Emergency medical treatment due to the occurrence of psychotic symptoms following cannabis use^15^	Acute cannabis-induced psychosis following the administration of oral cannabis^85^	Occurrence of acute psychotic episode during the trial^31^

### Predictors of cannabis-associated psychotic symptoms

Assessing predictors of CAPS, we included 103 studies published between 1976 and 2023, corresponding to 80 independent samples (*n* = 170,158 non-overlapping individuals). In total, we extracted 381 Cohen’s *d* that were pooled in 44 separate meta-analytical models. A summary of all extracted study estimates is provided in Supplementary Table 4. Comparing the *P* values of the individual Cohen’s *d* to the original *P* values as reported in the studies revealed a high level of concordance (*r* = 0.96 *P* = 1.1 × 10^–79^), indicating that the conversion of the raw study estimates to a common metric did not result in a substantial loss of information. Comparing the results obtained from the data extracted by two researchers (T.S. and W.B.) identified virtually no inconsistencies when inspecting estimates of Cohen’s *d*, as obtained for severity of cannabis use on CAPS (*d*_DIFF_ = 0, where *d*_DIFF_ = *d*_TS_ *–d* _WB_), gender (*d*_DIFF_ = 0), administration of (placebo controlled) medicinal cannabis (*d*_DIFF_ = 0.003), psychosis liability (*d*_DIFF_ = 0), and administration of a single dose of THC (*d*_DIFF_ = 0).

**Fig. 5:**
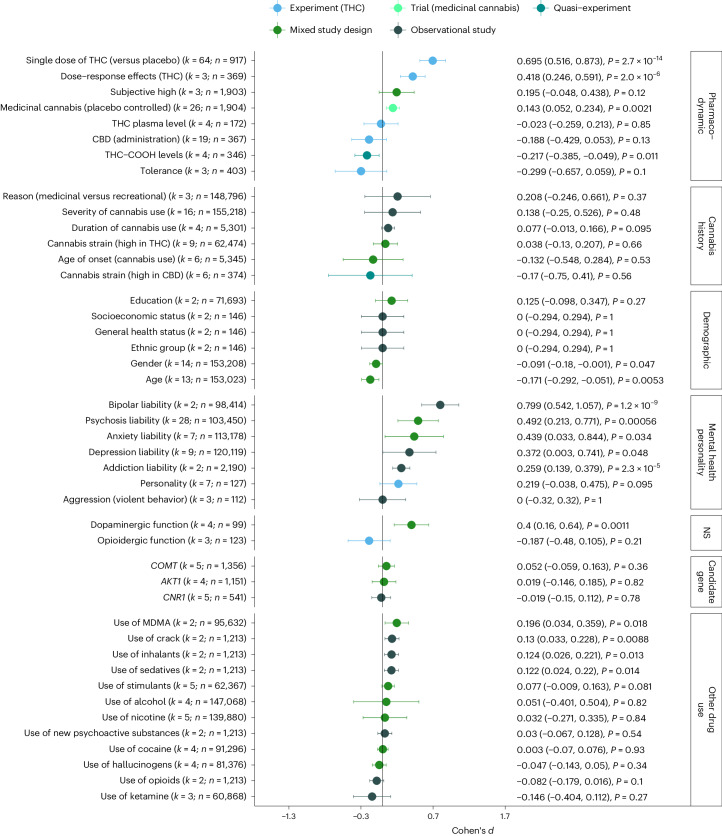
Predictors of cannabis-associated psychotic symptoms. Summary of pooled Cohen’s *d*, the corresponding 95% confidence intervals, and *P* values (two-sided, uncorrected for multiple testing). Positive estimates of Cohen’s *d* indicate increases in CAPS in response to the assessed predictor. Details regarding the classification and interpretation of each predictor are provided in the Supplementary Information. The reference list of all studies included in this figure is provided in Supplementary Table 4. NS, neurotransmission.

Figure 5 summarizes the results obtained from the meta-analytical models. We examined whether CAPS was predicted by the pharmacodynamic properties of cannabis, a person’s cannabis use history, demographic factors, mental health/personality traits, neurotransmitters, genetics, and use of other drugs: With respect to the pharmacodynamic properties of cannabis, the largest effect on CAPS severity was present for a single dose of THC (*d* = 0.7, 95% CI: 0.52, 0.87) as administered in experimental studies, followed by a significant dose–response effect of THC on CAPS (*d* = 0.42, 95% CI: 0.25, 0.59, that is, tested as moderation effects of THC dose in experimental studies). When tested in medicinal randomized controlled trials, cannabis products significantly increased symptoms of CAPS (*d* = 0.14, 95% CI: 0.05, 0.23), albeit by a smaller magnitude. Protective effects were present for low THC/COOH levels (*d* = −0.22, 95% CI: −0.39, −0.05, that is, the inactive metabolite of cannabis), but not for the THC/CBD (cannabidiol) ratio (*d* = −0.19, 95% CI: −0.43, 0.05, *P* = 0.13).

Less clear were the findings with respect to the cannabis use history of the participants and its effect on CAPS. Here, neither young age of onset of cannabis use nor high-frequency use of cannabis or the preferred type of cannabis (strains high in THC, strains high in CBD) was associated with CAPS. The only demographic factors that significantly predicted CAPS were age (*d* = −0.17, 95% CI: −0.292, −0.050) and gender (−0.09, 95% CI: −0.180, −0.001), indicating that younger and female cannabis users report higher levels of CAPS compared with older and male users. With respect to mental health and personality, the strongest predictors for CAPS were diagnosis of bipolar disorder (*d* = 0.8, 95% CI: 0.54, 1.06)) and psychosis liability (*d* = 0.49, 95% CI: 0.21, 0.77), followed by mood problems (anxiety *d* = 0.44, 95% CI: 0.03, 0.84; depression *d* = 0.37, 95% CI: 0.003, 0.740) and addiction liability (*d* = 0.26, 95% CI: 0.14, 0.38). Summarizing the evidence from studies looking at neurotransmitter functioning showed that increased dopamine activity significantly predicted CAPS (*d* = 0.4, 95% CI: 0.16, 0.64) (for example, reduced CAPS following administration of D2 blockers such as olanzapine^28^ or haloperidol^29^). By contrast, alterations in the opioid system did not reduce risk of CAPS. Similarly, none of the assessed candidate genes showed evidence of altering response to cannabis. Finally, out of 11 psychoactive substances with available data, only use histories of MDMA (3,4-methyl enedioxy methamphetamine) (*d* = 0.2, 95% CI: 0.03, 0.36), crack (*d* = 0.13, 95% CI: 0.03, 0.23), inhalants (*d* = 0.12, 95% CI: 0.03, 0.22), and sedatives (*d* = 0.12, 95% CI: 0.02, 0.22) linked to increases in CAPS.

Most of the meta-analytical models showed considerable levels of heterogeneity (*I*^*2*^ > 80%; Supplementary Table 5), notably when summarizing findings from observational studies (for example, severity of cannabis use: *I*^*2*^ = 98%, age of onset of cannabis use: *I*^*2*^ = 98%), highlighting that the individual effect estimates varied substantially across studies. By contrast, lower levels of heterogeneity were present when pooling evidence from experimental and medicinal cannabis studies (for example, effects of medicinal cannabis: *I*^*2*^ = 18%; THC dose–response effects: *I*^*2*^ = 37%). While risk of publication bias was indicated for four of the meta-analytical models (Egger’s test *P* < 0.05) (Supplementary Fig. 3), an inspection of trim-and-fill adjusted estimates did not alter the conclusions for (1) administration of a single dose of THC (*P*_Egger_ < 0.0001, *d*_unadjusted_ = 0.7, *d*_trim-and-fill_ = 0.49), (2) CBD administration (*P*_Egger_ = 0.0001, *d*_unadjusted_ = −0.19, *d*_trim-and-fill_ = −0.14, both *P* < 0.05), psychosis liability (*P*_Egger_ = 0.025, *d*_unadjusted_ = 0.49, *d*_trim-and-fill_ = 0.49), and (3) diagnosis of depression (*P*_Egger_ = 0.019, *d*_unadjusted_ = 0.37, *d*_trim-and-fill_ = 0.54). Outliers were identified for seven meta-analytical models (Supplementary Fig. 4). Removing outliers from the models did not substantially alter the conclusions drawn from the models, as indicated for age (*d* = −0.18, *d*_corr_ = −0.14, both *P* < 0.05); anxiety (*d* = 0.61, *d*_corr_ = 0.47, both *P* < 0.05), severity of cannabis use (*d* = 0.19, *d*_corr_ = 0.25, both *P* > 0.05), depression (*d* = 0.41, *d*_corr_ = 0.25, both *P* > 0.05), gender (*d* = −0.09, *d*_corr_ = −0.12, both *P* < 0.05), psychosis liability (*d* = 0.49, *d*_corr_ = 0.43, both *P* < 0.05), and administration of a single dose of THC (*d* = 0.6, *d*_corr_ = 0.56, both *P* < 0.05). Sensitivity checks assessing whether Cohen’s *d* changes as a function of within-subject correlation coefficient highlighted that the results were highly concordant (Supplementary Fig. 6). Minor deviations from the main analysis were present for the effects of a single dose of THC (*d*_*r*=0.3_ = 0.64 versus *d*_*r*=0.5_ = 0.69 versus *d*_*r*=0.7_ = 0.77) and dose–response effects of THC (*d*_*r*=0.3_ = 0.45 versus *d*_*r*=0.5_ = 0.42 versus *d*_*r*=0.7_ = 0.39), but this did not alter the interpretation of the findings.

**Fig. 6:**
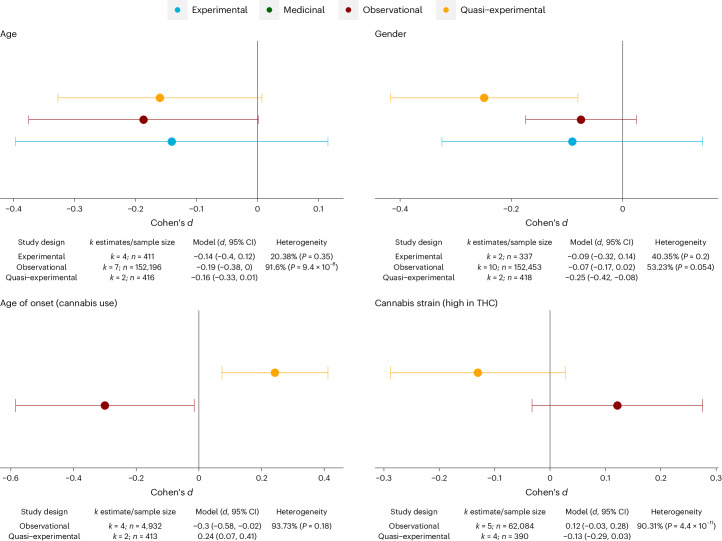
Triangulation of findings across different study designs. Pooled estimates of Cohen’s d when estimated separately for each of the different study designs. The *I*^*2*^ statistic (scale of 0 to 100) indexes the level of heterogeneity across the estimates included in the meta-analysis.

Finally, we assessed consistency of findings for predictors examined in more than one of the different study designs (observational, experimental, and medicinal cannabis studies), as illustrated for four meta-analytical models in Fig. 6 (see Supplementary Fig. 7 for the complete set of results). Triangulating the results highlighted that consistency with respect to the direction of effects was particularly high for age (*d*_Experiments_ = −0.14 versus *d*_Observational_ = −0.19 versus *d*_Quasi-Experimental_ = −0.16) and gender (*d*_Experiments_ = −0.09 versus *d*_Observational_ = −0.07 versus *d*_Quasi-Experimental_ = −0.25) on CAPS. By contrast, little consistency across the different study designs was present with respect to cannabis use histories, notable age of onset of cannabis use (*d*_Observational_ = −0.3 versus *d*_Quasi-Experimental_ = 0.24), and use of high-THC cannabis (*d*_Observational_ = 0.12 versus *d*_Quasi-Experimental_ = −0.13).

## Discussion

In this work, we examined rates and predictors of acute CAPS by synthesizing evidence from three distinct study designs: observational research, experimental studies administering THC, and studies testing medicinal cannabis products. Our results led to a number of key findings regarding the risk of CAPS in individuals exposed to cannabis. First, significant rates of CAPS were reported by all three study designs. This indicates that risk of acute psychosis-like symptoms exists after exposure to cannabis, irrespective of whether it is used recreationally, administered in controlled experiments, or prescribed as a medicinal product. Second, rates of CAPS vary across the different study designs, with substantially higher rates of CAPS in observational and experimental samples than in medicinal cannabis samples. Third, not every individual exposed to cannabis is equally at risk of CAPS as the interplay between individual differences and the pharmacological properties of the cannabis likely play an important role in modulating risk. In particular, risk appears most amplified in vulnerable individuals (for example, young age, pre-existing mental health problems) and increases with higher doses of THC (as shown in experimental studies).

### Rates of cannabis-associated psychotic symptoms

Summarizing the existing evidence on rates of CAPS, we find that cannabis can acutely induce CAPS in a subset of cannabis-exposed individuals, irrespective of whether it is used recreationally, administered in controlled experiments, or prescribed as a medicinal product. Importantly, rates of CAPS varied substantially across the designs. More specifically, similar rates of CAPS were reported by observational and experimental evidence (around 19% and 21% in cannabis-exposed individuals, respectively), while considerably lower rates of CAPS were documented in medicinal cannabis samples (between 1% and 2%).

A number of factors likely contribute to the apparently different rates of CAPS across the three study designs. First, rates of CAPS are not directly comparable as different, design-specific measures were used: in observational/experimental research, CAPS is typically defined as the occurrence of transient cannabis-induced psychosis-like symptoms, whereas medicinal trials screen for CAPS as the occurrence of first-rank psychotic symptoms, often resulting in treatment discontinuation.^20,30,31^ As such, transient CAPS may indeed occur commonly in cannabis-exposed individuals (as evident in the higher rates in observational/experimental research), while risk of severe CAPS requiring medical attention is less frequently reported (resulting in lower reported rates in medicinal cannabis samples). This converges with our meta-analytic results, showing that severe CAPS (full psychotic episode) may occur in about 1 in 200 (0.5%) cannabis users. Another key difference between medicinal trials and experimental/observational research lies in the demographic profile of participants recruited into the studies. For example, individuals taking part in medicinal trials were substantially older (mean age: 48 years) compared with subjects taking part in observational or experimental studies (mean age: 24 and 25 years, respectively). As such, older age may have buffered some of the adverse effects reported by adolescent individuals. Finally, cannabis products used in medicinal trials contain noticeable levels of CBD (for example, Sativex, with a THC/CBD ratio of approximately 1:1), a ratio different from that typically found in street cannabis (for example, >15% THC and <1% CBD^32^) and in the experimental studies included in our meta-analyses (pure THC). As such, the use of medicinal cannabis (as opposed to street cannabis) may constitute a somewhat safer option. However, the potentially protective effects of CBD in this context require further investigation as we did not find a consistent effect of CBD co-administration on THC-induced psychosis-like symptoms. While earlier experimental studies included in our work were suggestive of protective effects of CBD,^33–35^ two recent studies did not replicate these findings.^36,37^

Interestingly, lower but significant rates of CAPS were also observed in placebo groups assessed as part of THC-challenge studies (%_THC_ = 25% versus %_placebo_ = 11%) and medicinal cannabis trials (%_THC_ = 3% versus %_placebo_ = 1%), highlighting that psychotic symptoms occur not only in the context of cannabis exposure. This is in line with the notion that cannabis use can increase risk of psychosis but appears to be neither a sufficient nor necessary cause for the emergence of psychotic symptoms.^38^

### Predictors of CAPS

Summarizing evidence on predictors of CAPS, we found that individual vulnerabilities and the pharmacological properties of cannabis both appear to play an important role in modulating risk. Regarding the pharmacological properties of cannabis, evidence from experimental studies showed that the administration of THC increases risk of CAPS, both in a single-dose and dose-dependent manner. Given the nature of the experimental design, these effects are independent of potential confounders that bias estimates obtained from observational studies. More challenging to interpret are therefore findings on individual cannabis use histories (for example, frequency/severity of cannabis use, age of onset of use, preferred cannabis strain) as assessed in observational studies. Contrary to evidence linking high-frequency and early-onset cannabis use to long-term risk of psychosis,^39^ none of these factors associated with CAPS in our study. This discrepancy may indicate that cumulative effects of THC exposure are expressed differently for long-term risk of psychosis and acute CAPS: while users accustomed to cannabis may show a more blunted acute response as a result of tolerance, they are nevertheless at a higher risk of developing the clinical manifestation of psychosis in the long run.^38^

We also tested a number of meta-analytical models for predictors tapping into demographic and mental health dimensions. Interestingly, among the assessed demographic factors, only age and gender associated with CAPS, with younger and female individuals reporting increased levels of CAPS. Other factors often linked to mental health, such as education or socioeconomic status, were not related to CAPS. Concerning predictors indexing mental health, we found converging evidence showing that a predisposition to psychosis increased the risk of experiencing CAPS. In addition, individuals with other pre-existing mental health vulnerabilities (for example, bipolar disorder, depression, anxiety, addiction liability) also showed a higher risk of CAPS, indicating that risk may stem partly from a common vulnerability to mental health problems.

These findings align with findings from studies focusing on the biological correlates of CAPS, showing that increases in dopamine activity, a neurotransmitter implicated in the etiology of psychosis,^40^ altered sensitivity to cannabis. By contrast, none of the a priori selected candidate genes (chosen mostly to index schizophrenia liability) modulated risk of CAPS. This meta-analytic finding is coherent with results from the largest available genome-wide association study on schizophrenia,^41^ where none of the candidate genes reached genome-wide significance (*P* < 5 × 10^−8^) (Supplementary Information). Instead, as for any complex trait, genetic risk underlying CAPS is likely to be more polygenic in nature, possibly converging on pathways as yet to be identified. As such, genetic testing companies that screen for the aforementioned genetic variants to provide their customers with an individualized risk profile (such as the Cannabis Genetic Test offered by Lobo Genetics (https://www.lobogene.com)) are unlikely to fully capture the genetic risk underlying CAPS. Similarly, genetic counseling programs targeting specifically *AKT1* allele carriers in the context of cannabis use^42^ may be only of limited use when trying to reduce cannabis-associated harms.

### Implications for research on cannabis use and psychosis

This work has a number of implications for future research avenues. First, experimental studies administering THC constitute the most stringent available causal inference method when studying risk of CAPS. Future studies should therefore capitalize on experimental designs to advance our understanding of the acute pharmacological effects of cannabis, in terms of standard cannabis units,^43^ dose–response risk profiles,^44^ the interplay of different cannabinoids,^44,45^ and building on recent work.

Despite the value of experimental studies in causal inference, observational studies are essential to identify predictors of CAPS that cannot be experimentally manipulated (for example, age, long-term/chronic exposure to cannabis) and to strengthen external validity. However, a particular challenge for inference from observational studies results from bias due to confounding and reverse causation. Triangulating and comparing findings across study designs can therefore help to identify potential sources of bias that are specific to the different study designs.^46^ For example, we observed that, despite THC dosing being robustly associated with CAPS in experimental studies, we did not find an association between cannabis use patterns (for example, high-THC cannabis strain) in observational and quasi-observational studies. This apparent inconsistency may result from THC effects that are blunted by long-term, early-onset and heavy cannabis use. For other designs, reverse causation may bias the association between cannabis use patterns and CAPS: as individuals may reduce cannabis consumption as a result of adverse acute effects,^47^ the interpretation of cross-sectional estimates concerning different cannabis exposure and risk of CAPS is particularly challenging. Future observational studies should therefore exploit more robust causal inference methods (for example, THC administration in naturalistic settings^48^ or within-subject comparisons controlling for time-invariant confounds^49^) to better approximate the experimental design. In particular, innovative designs that can provide a higher temporal resolution on cannabis exposures and related experiences (for example, experience sampling,^50^ assessing daily reactivity to cannabis^51^) are a valuable addition to the causal inference toolbox for cannabis research. Applying genetically informed causal inferences such as Mendelian randomization analyses^52^ can further help to triangulate findings, which would be possible once genome-wide summary results for both different cannabis use patterns and CAPS become available.

With respect to medicinal trials, it is important to note that an assessment of CAPS has not been a primary research focus. Although psychotic events are recognized as a potential adverse reaction to medicinal cannabis,^53^ data on CAPS are rarely reported by medicinal trials, considering that only about 20% of medicinal cannabis randomized controlled trials screen for psychosis as a potential adverse effects.^5^ As such, trials should systematically monitor CAPS, in addition to longer-term follow-ups assessing the risk of psychosis as a result of medicinal cannabis use. In particular, the use of validated instruments designed to capture more-subtle changes in CAPS should be included in trials to more adequately assess adverse reactions associated with medicinal cannabis products.

Second, with respect to factors associated with risk of CAPS, we find that these are similar to factors associated with onset of psychosis, notably pre-existing mental health vulnerabilities,^54^ dose–response effects of cannabis,^55^ and young age.^12^ The key question deserving further attention is therefore whether CAPS constitutes, per se, a risk maker for long-term psychosis. Preliminary evidence found that in individuals with recent-onset psychosis, 37% reported to have experienced their first psychotic symptoms during cannabis intoxication.^56^ Future longitudinal evidence building on this is required to determine whether subclinical cannabis-associated psychotic symptoms can help to identify users at high risk of developing psychosis in the long run. Follow-up research should also examine longitudinal trajectories of adverse cannabis-induced experiences and the distress associated with these experiences, given research suggesting that high levels of distress/persistence may constitute a marker of clinical relevance of psychotic-like experiences.^57^ While few studies have explored this question in the context of CAPS, there is, for example, evidence suggesting that the level of distress caused by acute adverse reactions to cannabis may depend on the specific symptom dimension.^58^ Here the highest levels of distress resulted from cannabis-associated paranoia and anxiety, rather than cannabis-associated hallucinations or experiences tapping into physical sensations (for example, body humming, numbness). In addition, some evidence highlights the re-occurring nature of CAPS in cannabis-exposed individuals.^22,58^ Further research focusing on individuals with persisting symptoms of CAPS may therefore help to advance our knowledge concerning individual vulnerabilities underlying the development of long-term psychosis in the context of cannabis use.

Importantly, our synthesizing analysis is not immune to the sources of bias that exist for the different study designs, and our findings should therefore be considered in light of the aforementioned limitations (for example, residual confounding or reverse causation in observational studies, limited external validity in experimental studies). Nevertheless, comparing findings across the different study designs allowed us to pin down areas of inconsistency, which existed mostly with regard to cannabis-related parameters (for example, age of onset, frequency of use) and CAPS. In addition, we observed large levels of heterogeneity among most meta-analysis models, highlighting that study-specific findings may vary as a result of different sample characteristics and study methodologies. Future studies aiming to further discern potential sources of variation such as study design features (for example, treatment length in medicinal trials, route of THC administration in experimental studies), statistical modeling (for example, the type of confounding factors considered in observational research), and sample demographics (for example, age of the participants, previous experience with cannabis) are therefore essential when studying CAPS.

## Conclusions

Our results demonstrate that cannabis can induce acute psychotic symptoms in individuals using cannabis for recreational or medicinal purposes. Some individuals appear to be particularly sensitive to the adverse acute effects of cannabis, notably young individuals with pre-existing mental health problems and individuals exposed to high levels of THC. Future studies should therefore monitor more closely adverse cannabis-related outcomes in vulnerable individuals as these individuals may benefit most from harm-reduction efforts.

## Methods

### Systematic search

A systematic literature search was performed in three databases (MEDLINE, EMBASE, and PsycInfo) following the PRISMA guidelines.^59^ The final search was conducted on 6 December 2023 using 26 search terms indexing cannabis/THC and 20 terms indexing psychosis-like outcomes or cannabis-intoxication experiences (see Supplementary Information for a complete list of search terms). Search terms were chosen on the basis of terminology used in studies assessing CAPS, including observational studies (self-reported cannabis-induced psychosis-like experiences), THC-challenge studies (testing change in psychosis-like symptoms following THC administration), and medicinal studies testing the efficacy and safety of medicinal cannabis products (adverse events related to medicinal cannabis). Before screening the identified studies for inclusion, we removed non-relevant article types (reviews, case reports, comments, guidelines, editorials, letters, newspaper articles, book chapters, dissertations, conference abstracts) and duplicates using the R package revtools^60^. A senior researcher experienced in meta-analyses on cannabis use (T.S.) then reviewed all titles and abstracts for their relevance before conducting full-text screening. To reduce the risk of wrongful inclusion at the full-text screening stage, 10% of the articles selected for full-text screening were cross-checked for eligibility by a second researcher (E.M.).

### Data extraction

We included all study estimates that could be used to derive rates of CAPS (the proportion of cannabis-exposed individuals reporting CAPS) or effect sizes (Cohen’s *d*) for factors predicting CAPS. CAPS was defined as the occurrence of hallucinations, paranoia, and/or delusions during cannabis intoxication. These symptom-level items have been identified as the most reliable self-report measures screening for psychosis when validated against clinical interview measures.^61^ Table 1 provides examples of CAPS as measured across the three different study designs. In brief, from observational studies, we extracted data if CAPS was assessed in cannabis-exposed individuals on the basis of self-report measures screening for subjective experiences while under the influence of cannabis. From experimental studies administering THC, CAPS was measured as the degree of psychotic symptom change in response to THC, either estimated from a between-subject (placebo groups versus THC group) or within-subject (pre-THC versus post-THC assessment) comparison. We also included data from natural experiments (referred to as quasi-experimental studies hereafter), where psychosis-like experiences were monitored in recreational cannabis users before and after they consumed their own cannabis products.^23,62^ Finally, with respect to trials testing the efficacy and/or safety of medicinal cannabis products containing THC, we extracted data on adverse events, including the occurrence of psychosis, hallucinations, delusions, and/or paranoia during treatment with medicinal cannabis products. Medicinal studies that tested the effects of cannabis products not containing THC (for example, CBD only, olorinab, lenabasum) were not included.

For 10% of the included studies, data on rates and predictors of CAPS were extracted by a second researcher (W.B.), and agreement between the two extracted datasets was assessed by comparing the pooled estimates on rates and predictors of CAPS. In addition, following recommendations for improved reproducibility and transparency in meta-analytical works,^63^ we provide all extracted data, the corresponding analytical scripts, and transformation information in the study repository.

### Statistical analysis

#### Rates of CAPS

We extracted the raw estimates of rates of CAPS as reported by observational, experimental, and medicinal cannabis studies. Classification of CAPS differs across the three study designs. In observational studies, occurrence of CAPS is typically defined as the experience of psychotic-like symptoms while under the influence of cannabis. In experimental studies administering THC, CAPS is commonly defined as a clinically significant change in psychotic symptom severity (for example, ≥3 points increase in Positive and Negative Syndrome Scale positive scores following THC^33^). Finally, in medicinal cannabis samples, a binary measure of CAPS indicates whether psychotic symptoms occurred as an adverse event throughout the treatment with medicinal cannabis products. We derived rates of CAPS (*R*_CAPS_ = *X*_Count of CAPS_/*N*_Sample size_) and the corresponding confidence intervals using the function BinomCI and the Clopper–Pearson method as implemented in the R package DescTools.^64^ To estimate the pooled proportions, we fitted random-effects models or multilevel random-effects models as implemented in the R package metafor.^65^ Multilevel random-effects models were used whenever accounting for non-independent sampling errors was necessary (further described in the following). Risk of publication bias was assessed using Peters’ test^66^ and funnel plots and, if indicated (*P*_Peters_ < 0.05), corrected using the trim-and-fill method (Supplementary Methods).

#### Predictors of CAPS

To derive the pooled effects of factors predicting CAPS, we converted study estimates to the standardized effect size Cohen’s *d* as a common metric. For studies reporting mean differences, two formulas were used for the conversion. First, for studies reporting mean differences from between-subject comparisons (independent samples), we used the following formula:

$$d=\,\frac{{M}_{{\mathrm{E}}}-\,{M}_{{\mathrm{C}}}}{{{{\mathrm{SD}}}}_{{\mathrm{P}}}}$$

where *M*_E_ and *M*_C_ are the mean scores on a continuous scale (severity of CAPS), reported for individuals exposed (*M*_E_) and unexposed (*M*_C_) to a certain risk factor (for example, cannabis users with pre-existing mental health problems versus cannabis users without pre-existing mental health problems). The formula used to derive the pooled standard deviations, SD_P_, and the variance of Cohen’s *d* are listed in the Supplementary Methods. Second, an extension of the preceding formula was used to derive Cohen’s *d* from within-subject comparisons, comparing time-point one (*M*_T1_) with time-point two (*M*_T2_).The formula takes into account the dependency between the two groups:^67^

$$d=\frac{{M}_{{\mathrm{T}}1}-{M}_{{\mathrm{T}}2}}{\sqrt{{{{\mathrm{SD}}}}_{{\mathrm{T}}1}^{2}+{{{\mathrm{SD}}}}_{{\mathrm{T}}2}^{2}-2r\;{{{\mathrm{SD}}}}_{{\mathrm{T}}1}{{{\mathrm{SD}}}}_{{\mathrm{T}}2}}}$$

where *r* indexes the correlation between the pairs of observations, such as the correlation between the pre- and post-THC condition in the same set of individuals for a particular outcome measure. The correlation coefficient was set to be *r* = 0.5 for all studies included in the meta-analysis, on the basis of previous research.^13^ We also assessed whether varying within-person correlation coefficients altered the interpretation of the results by re-estimating the pooled Cohen’s *d* for predictors of CAPS for two additional coefficients (*r* = 0.3 and *r* = 0.7). The results were then compared with the findings obtained from the main analysis (*r* = 0.5).

From experimental studies reporting multiple time points of psychosis-like experiences following THC administration (for example, refs. ^68–72^), we selected the most immediate time point following THC administration. Of note, whenever studies reported test statistics instead of means (for example, *t*-test or *F*-test statistics), the preceding formula was amended to accommodate these statistics. In addition, to allow for the inclusion of studies reporting metrics other than mean comparisons (for example, regression coefficients, correlations coefficients), we converted the results to Cohen’s *d* using existing formulas. All formulas used in this study are provided in the Supplementary Information. Whenever studies reported non-significant results without providing sufficient data to estimate Cohen’s *d (*for example, results reported only as *P* > 0.05*)*, we used a conservative estimate of *P* = 1 and the corresponding sample size as the input to derive Cohen’s *d*. Finally, if studies reported estimates in figures only, we used WebPlotDigitizer (https://automeris.io/WebPlotDigitizer) to extract the data. Since the conversion of estimates from one metric to another may result in loss of precision, we also extracted the original *P*-value estimates (whenever reported as numerical values) and assessed the level of concordance with the *P* values corresponding to the estimated Cohen’s *d*.

Next, a series of meta-analytical models were fitted, each pooling estimates of Cohen’s *d* that belonged to the same class of predictors (for example, estimates indexing the effect of dopaminergic function on CAPS; estimates indexing the effect of age on CAPS). A detailed description of the classification of the included predictors is provided in the Supplementary Methods. Cohen’s *d* estimates were pooled if at least two estimates were available for one predictor class, using one of the following models:

1. Aggregation models (pooling effect sizes coming from the same underlying sample)
2. Random-effects models (pooling effect sizes coming from independent samples)
3. Multilevel random-effects models (pooling effect sizes coming from both independent and non-independent samples)

Predictors that could not meaningfully be grouped were not included in meta-analytical models but are, for completeness, reported as individual study estimates in the Supplementary Information. Levels of heterogeneity for each meta-analytical model were explored using the *I*^*2*^ statistic,^73^ indexing the contribution of study heterogeneity to the total variance. Here, *I*^*2*^ > 30% represents moderate heterogeneity and *I*^*2*^ > 50% represents substantial heterogeneity. Risk of publication bias was assessed visually using funnel plots alongside the application of Egger’s test to test for funnel-plot asymmetry. This test was performed for meta-analytical models containing at least six effect estimates.^74^ The trim-and-fill^75^ method was used whenever risk of publication bias was indicated (*P*_Egger_ < 0.05). To assess whether outliers distorted the conclusions of the meta-analytical models, we applied leave-one-out and outlier analysis^76^ as implemented in the R package dmetar,^77^ where a pooled estimate was re-calculated after omitting studies that deviated from the pooled estimate. Further details on all applied sensitivity analyses are provided in the Supplementary Methods.

### Reporting summary

Further information on research design is available in the Nature Portfolio Reporting Summary linked to this article.

## Supplementary information

Supplementary Information Supplementary Figs. 1–7, Methods (literature search, estimation of Cohen’s *d*, classification of predictors of CAPS, analysis plan), and references.

Reporting Summary

Supplementary Tables Supplementary Tables 1–5.

## Data Availability

The data are publicly available via GitHub at github.com/TabeaSchoeler/TS2023_MetaCAPS.

## Code availability

All analytical code used to analyze, summarize, and present the data is accessible via GitHub at github.com/TabeaSchoeler/TS2023_MetaCAPS.
