# Supplementary material for: Assessing rates and predictors of cannabis-associated psychotic symptoms across observational, experimental and medical research
Source: Nat Ment Health. 2024 Jun 3;2(7):865–76. doi: 10.1038/s44220-024-00261-x (PMC11236708; doi:10.1038/s44220-024-00261-x)
Supplement: Supplementary file 1 — Supplementary Figs. 1–7, Methods (literature search, estimation of Cohen’s d, classification of predictors of CAPS, analysis plan), and references. [file 44220_2024_261_MOESM1_ESM.pdf]

nature mental health

Supplementary information https://doi.org/10.1038/s44220-024-00261-x

Assessing rates and predictors of cannabis-associated psychotic symptoms across observational, experimental and medical research

In the format provided by the authors and unedited

Supplement information

SMETHODS 2

LITERATURE SEARCH 2
SEARCH TERMS INCLUDED IN THE SYSTEMATIC SEARCH 2
ESTIMATION OF COHEN'S *D* 3
FORMULA USED TO DERIVE COHEN'S D 3
CLASSIFICATION OF PREDICTORS 4
CANDIDATE GENES AND SNP EFFECTS ON RISK OF SCHIZOPHRENIA 5
ANALYSIS 6
ESTIMATION OF POOLED EFFECT SIZE COHEN D 6

SRESULTS 7

SFIGURE 1. PRISMA FLOW CHART 7
PRISMA REPORTING CHECKLIST 8
SFIGURE 2. RATES OF CANNABIS-ASSOCIATED PSYCHOTIC SYMPTOMS ACROSS STUDY DESIGNS 10
SFIGURE 3. PETERS' TEST FOR PUBLICATION BIAS (RATES OF CAPS) 11
SFIGURE 4. EGGER'S TEST FOR PUBLICATION BIAS (PREDICTORS OF CAPS) 12
SFIGURE 5. LEAVE-ONE-OUT AND OUTLIER ANALYSIS 13
SFIGURE 6. COMPARISON OF ESTIMATES OF COHEN'S D OBTAINED FOR PREDICTORS OF CAPS, WITH VARYING LEVELS OF WITHING-SUBJECT CORRELATIONS 17
SFIGURE 7. TRIANGULATION OF FINDINGS ACROSS DIFFERENT STUDY DESIGNS 18
QUESTIONNAIRES USED TO ASSESS CANNABIS-ASSOCIATED PSYCHOTIC SYMPTOMS 19

SREFERENCES 20

sMethods

Literature search

We searched a number of databases previously used in meta-analysis on cannabis related effects, including MEDLINE, EMBASE and PsychInfo. Search terms indexing the outcome of interest were selected as follows: (1) validated measures assessing psychosis symptoms (e.g. Positive and Negative Syndrome Scale, PANSS) used in experimental and observational data, (2) psychosis symptoms as monitored in medical trials (i.e., adverse drug reactions including hallucinations and paranoia) and (3) broader terms used to describe outcomes related to psychosis.

Search terms included in the systematic search	
Cannabis term	Psychosis term
Hashis*	Psychosis
Hash	Psychotic
Skunk	Psychoses
Bhang	Psychotomimetic
Ganja	Community assessment of psychic experience scale
Ganjah	Positive and Negative Syndrome Scale
Hemp	Brief Psychiatric Rating Scale
Charas	Scale for the Assessment of Positive Symptoms
Cannabis	Psychotomimetic States Inventory
Marijuana	Cannabis Experience Questionnaire
Marihuana	Paranoia
Dronabinol	Hallucination(s)
Marinol	Delusion(s)
Levonantradol	Intoxicating
Tetrahydrocannabinol	Intoxication
Cesamet	Adverse event(s)
THC	Adverse drug reaction(s)
Nabiximols	Adverse effect(s)
Sativex	Adverse event(s)
JWH-018	Visual analogue scale(s)
Bedrobinol	
WIN,55	
CP55940	
Bedrocan	
Cannabinoid(s)	
Nabilone	

Estimation of Cohen's *d*

Formula used to derive Cohen's d

(1) Estimate Cohen's d from unpaired means

$$d = \frac{M_E - M_C}{SD_P} \qquad SD_P = \frac{\sqrt{SD_E^2 + SD_C^2}}{2}$$

$$Var_d = \frac{n_E + n_C}{n_E n_C} + \frac{d^2}{2(n_E + n_C)}$$

> M_E and M_C are the mean scores on a continuous measure, estimated for cannabis users exposed (n_E) to the risk factor of interest and unexposed controls (n_C)
> SD_P is the pooled standard deviation, estimated using the standard deviations corresponding to M_e and M_C
> Var_d is the variance of *d*
All formula are described in Borenstein (p.26, formula 4.18 – 4.21)[1]

Note. For studies not reporting means and standard deviations per group, *d* was derived using the reported t-statistic or F-statistic, based on the formula's included in the R package 'estimate.es' [cf. function tes() or fes(), respectively].

(2) Estimate Cohen's d from unstandardized linear regression coefficients[2]

$$d = \frac{\beta}{SE(\beta)}\sqrt{\frac{1}{n_E} + \frac{1}{n_C}}$$

Var_d was estimated using the 'ci.smd' function of the R-package MBESS
> β *is the unstandardized linear regression coefficient estimate divided by the corresponding standard error*
> *n indicates the sample size for* cannabis users exposed (n_E) to the risk factor of interest and controls (n_C)

(3) Estimate Cohen's d from paired means

$$d = \frac{M_E - M_C}{\sqrt{SD_E^2 - SD_C^2 - 2rSD_E SD_C}} \qquad Var_d = \left(\frac{1}{n} + \frac{d^2}{2n}\right)2(1-r)$$

> *r,* the correlation between pairs of observations (e.g., within-subject correlation between a pretest and posttest measure)
> M_E, the mean in the group exposed to the risk factor (e.g. administration of THC)
> M_C, the mean in the control group (e.g. placebo condition)
> SD_E, the standard in the group exposed to the risk factor (e.g. administration of THC)
> SD_C, the standard deviation in the control group (e.g. placebo condition)
> Var_d is the variance of *d*

Note. The equations can be found in Borenstein (p.29, Formula 4.28)[1]. If only t-test statistics were reported for mean differences between within-subject comparisons, we used the standard formula to estimate *d* (2) and estimated the confidence intervals using noncentrality parameters (ncp)[3].

(4) Estimate Cohen's d using effective sample sizes

$$N_{EFF} = \frac{(2N_W)}{(1-r)}$$

> N_W indexes the sample size from a within-subject design
> N_{EFF}, the effective sample size

From Maxwell & Delaney (2004, p. 561, formula 45)[4]. If no mean and SD estimates or t-test statistics were reported for within-subjects comparisons, we converted p-values, F-statistics or Chi-Square statistics to Cohen's *d* calculation including the effective sample size.

(5) Comparison between two effect sizes (Cohen's d)

$$d_{diff} = d_1 - d_2 \qquad Var_{diff} = Var_{d1} + Var_{d2}$$

To test differences between independent Cohen's *d*'s. From: Borenstein[5] (p. 156).

Var_d estimation for dependent Cohen's *d*'s:

$$Var_{diff} = Var_{d1} + Var_{d2} - 2r\sqrt{Var_{d1}}\sqrt{Var_{d2}}$$

Classification of predictors

Pharmacodynamic factors

- Single dose of THC = effect of a single dose of THC as administered in experimental studies, where a positive Cohen's *d* indexes increasing levels of CAPS as a result of THC administration.
- Subjective high = self-reported high as assessed in experimental studies following the administration of THC, where a positive Cohen's *d* links increasing levels of subjective high to more severe CAPS.
- Dose-response effects = dose-response effects of THC as tested in form of moderating effects in experimental studies administering multiple doses of THC. A positive Cohen's *d* indexes that higher doses of THC link to increases in levels of CAPS.
- Medical cannabis (placebo controlled) = evidence from placebo-controlled medical trials assessing the effect of medical cannabis products containing THC on CAPS. A positive Cohen's *d* indexes that the administration of medical cannabis products associates with increased levels of CAPS.
- THC-COOH levels = levels of THC-COOH as measured in blood or urine. A positive Cohen's *d* indexes an association between increasing THC-COOH and higher levels of CAPS.
- CBD pre-treatment = the effects of CBD administration in experimental studies testing the effects of THC, where a negative Cohen's *d* indexes reduced CAPS as a results of CBD co-administration/pre-treatment.
- Tolerance = defined as the level of THC in the body prior to the administration of THC as assessed in (quasi-)experimental studies. A negative Cohen's *d* indexes that increased levels of tolerance link to lower THC-induced CAPS.

Cannabis history

- Reason (medicinal vs. recreational) = comparison of levels of CAPS between users of (self-reported) recreational cannabis and users of medicinal cannabis, where a positive Cohen's *d* indicates higher levels of CAPS in recreational users.
- Cannabis strain (high THC) = comparison of individuals using more potent forms of cannabis to users of milder forms of cannabis. A positive Cohen's *d* indexes that higher levels of CAPS associate with the use of more potent cannabis.
- Severity of cannabis use = summary of evidence looking at different parameters of cannabis use patterns (e.g., high frequency use versus low frequency use, number of joints smoked per day). A positive Cohen's *d* reflects increasing levels of CAPS in individuals with a more heavy cannabis use pattern.
- Cannabis strain (high CBD) = comparison of individuals using CBD-rich forms of cannabis to users of other forms of cannabis.

- Age of onset (cannabis use) = estimates the relation between age of onset of cannabis use and levels of CAPS. A positive Cohen's *d* indexes older age of onset linking to more severe CAPS.

Demographic variables

- Gender = a positive Cohen's *d* indexes increased risk of CAPS for male individuals exposed to cannabis/THC.
- Age = a positive Cohen's *d* indexes that older age associates with higher levels of CAPS.
- Education = a positive Cohen's *d* indexes that more years spent in education associate with higher levels of CAPS

Neurotransmitter systems

A higher Cohen's *d* indexing that increased neurotransmitter activty links to increased risk of CAPS.

- Dopamine: In experimental studies on dopamine, dopamine activity was either manipulated by the administration of drugs reducing dopamine activity, including D2 blockers such as olanzapine, haloperidol, or was measured directly in positron emission tomography studies.
- Glutamate: Glutamine levels, measured as levels of glutamate-derived metabolites Glutamate (Glu) or Glutamate + Glutamine (Glx) using proton magnetic resonance spectroscopy.
- GABA studies used the Iomazenil to alter levels of GABA in the brain.
- Studies focusing of opioigergic agent used agents that lead to reductions (e.g., Naltrexone) or increases (e.g., Morphine) in opioid activity.

Mental health vulnerabilities

- A higher Cohen's *d* indicates that increasing mental health vulnerabilities link to increased risk of CAPS. Vulnerability was defined as 1) history of a clinical manifestation of a mental health condition (e.g., diagnosis of psychosis[6,7], bipolar disorder[8,9], anxiety disorder[9], history of a depressive episode[10]), 2) pre-existing at-risk mental state (e.g., clinical high-risk for psychosis[11], ultrahigh-risk state for psychosis[12]) 3) mental health liability in the general population (e.g., depression score[13], anxiety score[14], psychosis symptom severity prior THC administration[15]), family history (e.g., family history of addiction[14,16], schizophrenia[16], depression[13]) and genetic liability (e.g., polygenic scores for schizophrenia[17]).

Candidate genes and SNP effects on risk of schizophrenia				
Gene	rsID	Risk allele	Alternative allele	*p* ($GWAS_{Schz}$)*
COMT	rs4680	G	A	0.072
AKT1	rs2494732	C	T	0.06
AKT1	rs1130233	A	G	0.53
DAT1	3'UTR VNTR	9/9	10/10	
CNR1	rs806379	T	A	0.32
CNR1	rs1535255	C	A	0.23
CNR1	rs2023239	A	G	0.19
CNR1	rs1049353	G	A	0.068
CNR1	rs12720071	A	G	0.37
BDNF	rs6265	G	A	1.9e-05
FAAH	rs324420	A	C	0.89
p-value estimates extracted from schizophrenia GWAS ($n_{Effective}$=58,750)[18]				

Analysis

Estimation of pooled effect size Cohen D

Effect sizes were pooled together using one of the following models to pool together estimates of Cohen's *d*:

1) Aggregation models, to pool together effect sizes that were all derived from the same sample of participants. For that, we used the R package MAd and assumed r=0.5 to infer the correlation coefficient indexing the level of dependency.
2) Random effects models (REM) were used whenever all included estimates of Cohen's *d* were derived from independent study samples. We employed REM as implemented in the R package 'metafor'[19], keeping the default parameters and applying the Nelder-Mead optimizer for model fitting.
3) Multi-level random effects model (MREM), to pool together estimates of Cohen's *d* were some, but not all, came from the same underlying study sample. Here, we used the MREM model as implemented in the R package 'metafor' to account for dependency among estimates of Cohen's *d*. In this study, we employed a three-level MREM incorporating three sources of variation: (1) variation in effect sizes attributable to random sampling of effect sizes (Level 1, variance that is unique for each estimated Cohen's *d* per outcome), (2) variation in Cohen's *d* between outcomes within a single cohort (Level 2, variance that is common to all outcomes within a single cohort), and (3) variation in Cohen's *d* between different study cohorts (Level 3, variance that is common to all cohorts).

Test for publication bias

To test for publication bias when pooling together proportions (i.e., rates of CAPS), we used the weighted linear regression test proposed by Peters[20], which tests a linear regression model of the rates of CAPS on the inverse of the sample size, weighted by the variances. We used Egger's test to formally assess risk of publication bias for pooled estimates of Cohen's *d* (i.e., predictors of CAPS), which tests a linear regression model of the effect sizes on their standard errors weighted by their inverse variance. The tests were applied to all meta-analytical models containing at least six effect estimates[21]. Whenever publication bias was indicated ($p_{\text{Peters}} < 0.05$ / $p_{\text{Egger}}<0.05$), we used the trim-and-fill method[22], which obtains an estimate of the pooled effect size that is adjusted for publication bias. As this test is only available for random effects models (REM), we replaced the multilevel random effects models and re-ran the analysis using REM before adjusting for publication bias. To ensure independence of effect sizes, overlapping samples were excluded from the model.

Leave-one-out and outlier analysis

Between-study heterogeneity may have resulted from one or more studies with extreme effect estimates. We first re-calculated the pooled Cohen's d omitting one study at a time. To further assess if the meta-analytical models were significantly affected by outlying estimates among the included studies, we used the 'find.outliers' function as implemented in the R-Package dmetar[23]. Here, outliers were identified by comparing the individual study estimates to the pooled effect size. We then removed outlying study estimates and re-tested the meta-analytical model. The outlier analysis was applied to all REM models assessed for publication bias (cf. paragraph above).

sResults

sFigure 1. PRISMA Flow Chart

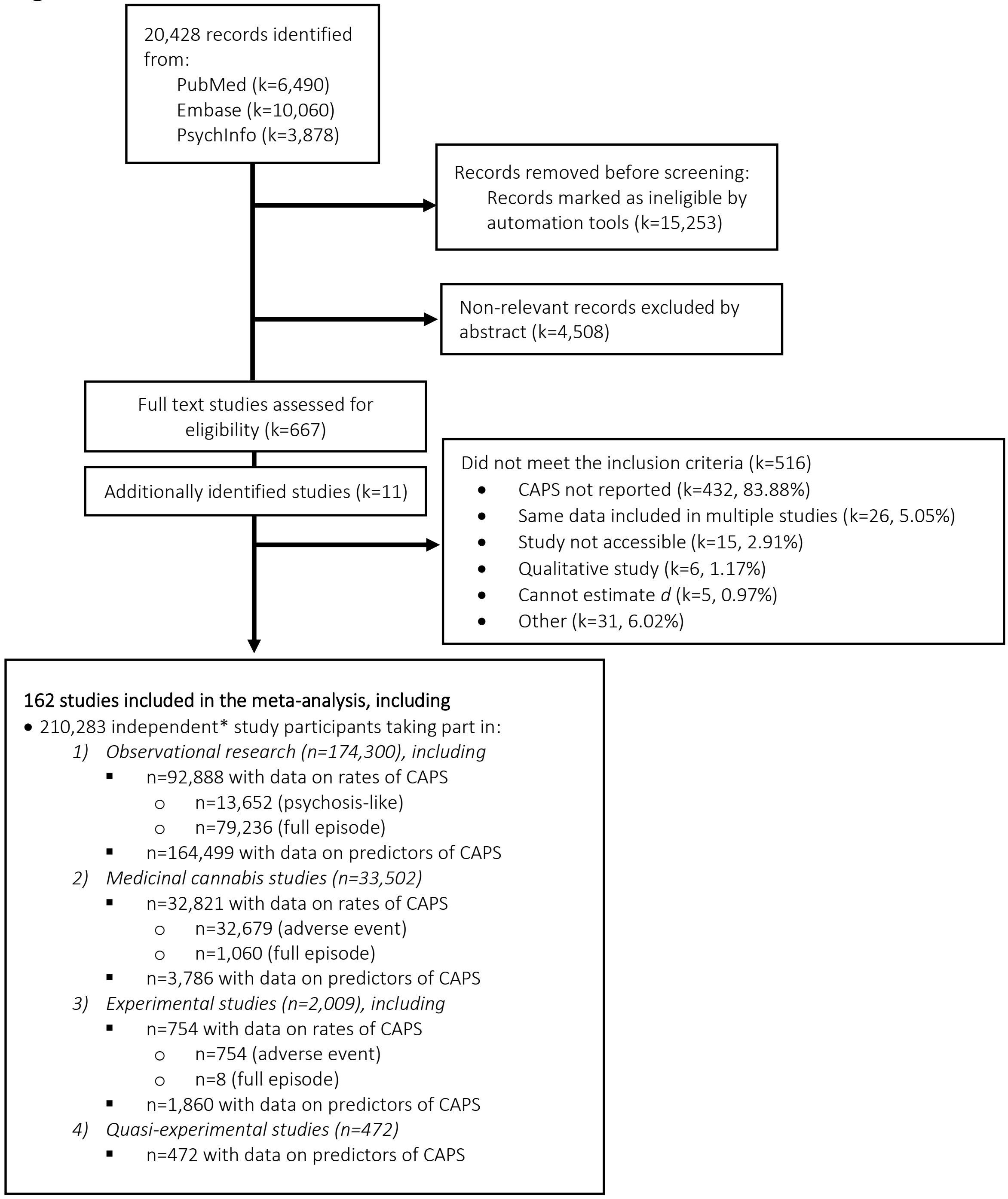

Flow chart as adapted from the Preferred Reporting Items for Systematic Reviews and Meta-Analyses (PRISMA) flowchart (http://www.prisma-statement.org/). *Independent study participants, defined as the maximum number of participants available for an underlying study sample assessed in one or more of the included studies.

PRISMA reporting checklist

Section and Topic	Item #	Checklist item	Location where item is reported
**TITLE**			
Title	1	Identify the report as a systematic review.	NA
**ABSTRACT**			
Abstract	2	See the PRISMA 2020 for Abstracts checklist.	2 (main)
**INTRODUCTION**			
Rationale	3	Describe the rationale for the review in the context of existing knowledge.	4 (main)
Objectives	4	Provide an explicit statement of the objective(s) or question(s) the review addresses.	4 (main)
**METHODS**			
Eligibility criteria	5	Specify the inclusion and exclusion criteria for the review and how studies were grouped for the syntheses.	15/16 (main) sMethods
Information sources	6	Specify all databases, registers, websites, organisations, reference lists and other sources searched or consulted to identify studies. Specify the date when each source was last searched or consulted.	15 (main)
Search strategy	7	Present the full search strategies for all databases, registers and websites, including any filters and limits used.	sMethods
Selection process	8	Specify the methods used to decide whether a study met the inclusion criteria of the review, including how many reviewers screened each record and each report retrieved, whether they worked independently, and if applicable, details of automation tools used in the process.	sMethods
Data collection process	9	Specify the methods used to collect data from reports, including how many reviewers collected data from each report, whether they worked independently, any processes for obtaining or confirming data from study investigators, and if applicable, details of automation tools used in the process.	15/16 (main) sMethods
Data items	10a	List and define all outcomes for which data were sought. Specify whether all results that were compatible with each outcome domain in each study were sought (e.g. for all measures, time points, analyses), and if not, the methods used to decide which results to collect.	16-18 (main) sMethods
	10b	List and define all other variables for which data were sought (e.g. participant and intervention characteristics, funding sources). Describe any assumptions made about any missing or unclear information.	sMethods
Study risk of bias assessment	11	Specify the methods used to assess risk of bias in the included studies, including details of the tool(s) used, how many reviewers assessed each study and whether they worked independently, and if applicable, details of automation tools used in the process.	15/16 (main) sMethods
Effect measures	12	Specify for each outcome the effect measure(s) (e.g. risk ratio, mean difference) used in the synthesis or presentation of results.	17/18 (main) sMethods
Synthesis methods	13a	Describe the processes used to decide which studies were eligible for each synthesis (e.g. tabulating the study intervention characteristics and comparing against the planned groups for each synthesis (item #5)).	NA
	13b	Describe any methods required to prepare the data for presentation or synthesis, such as handling of missing summary statistics, or data conversions.	17/18 sMethods
	13c	Describe any methods used to tabulate or visually display results of individual studies and syntheses.	NA
	13d	Describe any methods used to synthesize results and provide a rationale for the choice(s). If meta-analysis was performed, describe the model(s), method(s) to identify the presence and extent of statistical heterogeneity, and software package(s) used.	17-19 sMethods
	13e	Describe any methods used to explore possible causes of heterogeneity among study results (e.g. subgroup analysis, meta-regression).	17-19 sMethods
	13f	Describe any sensitivity analyses conducted to assess robustness of the synthesized results.	17-19 sMethods
Reporting bias assessment	14	Describe any methods used to assess risk of bias due to missing results in a synthesis (arising from reporting biases).	NA
Certainty assessment	15	Describe any methods used to assess certainty (or confidence) in the body of evidence for an outcome.	NA
**RESULTS**			
Study selection	16a	Describe the results of the search and selection process, from the number of records identified in the search to the number of studies included in the review, ideally using a flow diagram.	sFigure 1

Section and Topic	Item #	Checklist item	Location where item is reported
	16b	Cite studies that might appear to meet the inclusion criteria, but which were excluded, and explain why they were excluded.	sFigure 1
Study characteristics	17	Cite each included study and present its characteristics.	sTable 2/3
Risk of bias in studies	18	Present assessments of risk of bias for each included study.	NA
Results of individual studies	19	For all outcomes, present, for each study: (a) summary statistics for each group (where appropriate) and (b) an effect estimate and its precision (e.g. confidence/credible interval), ideally using structured tables or plots.	sTable 2/3
Results of syntheses	20a	For each synthesis, briefly summarise the characteristics and risk of bias among contributing studies.	6 (main) sFigure 3/4/5
	20b	Present results of all statistical syntheses conducted. If meta-analysis was done, present for each the summary estimate and its precision (e.g. confidence/credible interval) and measures of statistical heterogeneity. If comparing groups, describe the direction of the effect.	sTable 5 Figure 2/3/4
	20c	Present results of all investigations of possible causes of heterogeneity among study results.	7/8 (main)
	20d	Present results of all sensitivity analyses conducted to assess the robustness of the synthesized results.	8 (main)
Reporting biases	21	Present assessments of risk of bias due to missing results (arising from reporting biases) for each synthesis assessed.	8 (main)
Certainty of evidence	22	Present assessments of certainty (or confidence) in the body of evidence for each outcome assessed.	Figure 3/4/5/6
**DISCUSSION**			
Discussion	23a	Provide a general interpretation of the results in the context of other evidence.	10-14
	23b	Discuss any limitations of the evidence included in the review.	10-14
	23c	Discuss any limitations of the review processes used.	10-14
	23d	Discuss implications of the results for practice, policy, and future research.	10-14
**OTHER INFORMATION**			
Registration and protocol	24a	Provide registration information for the review, including register name and registration number, or state that the review was not registered.	NA
	24b	Indicate where the review protocol can be accessed, or state that a protocol was not prepared.	NA
	24c	Describe and explain any amendments to information provided at registration or in the protocol.	NA
Support	25	Describe sources of financial or non-financial support for the review, and the role of the funders or sponsors in the review.	20
Competing interests	26	Declare any competing interests of review authors.	20
Availability of data, code and other materials	27	Report which of the following are publicly available and where they can be found: template data collection forms; data extracted from included studies; data used for all analyses; analytic code; any other materials used in the review.	2-

sFigure 2. Rates of cannabis-associated psychotic symptoms across study designs

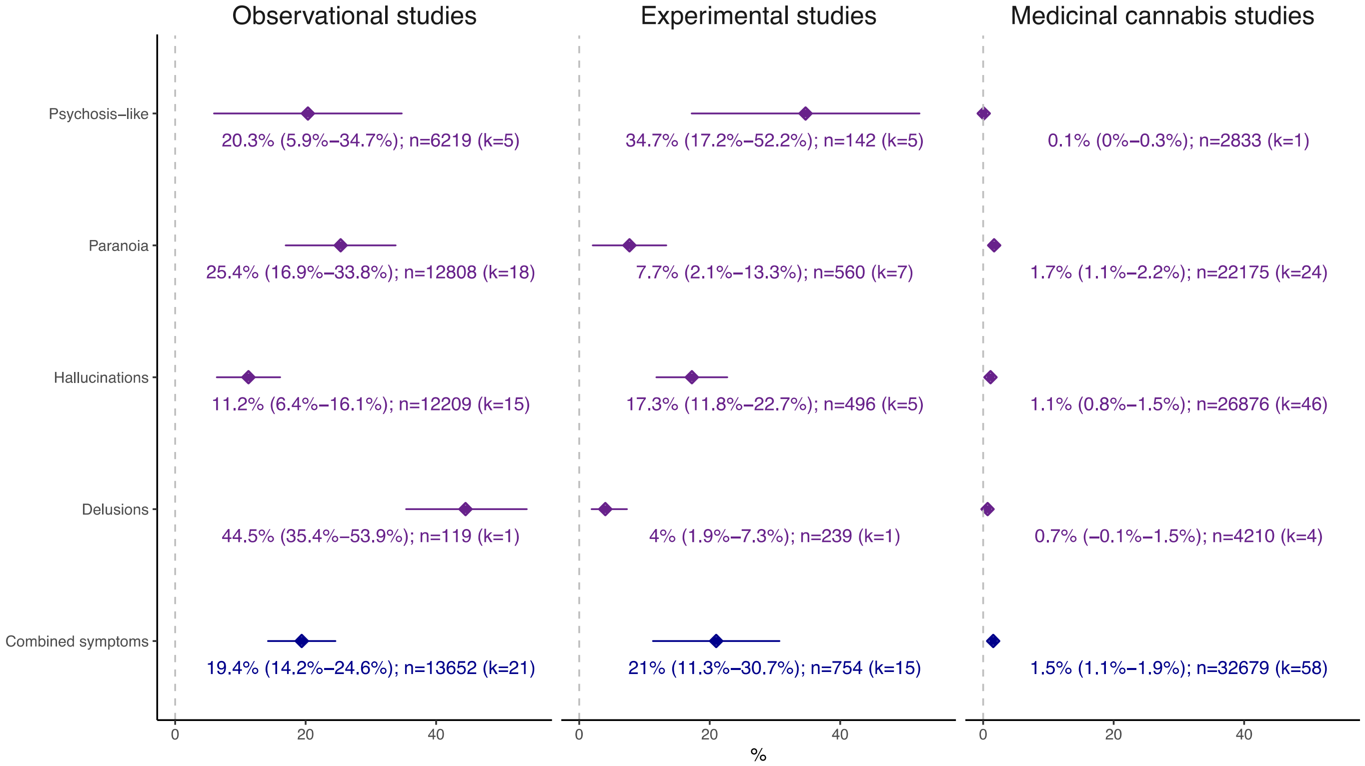

The figure shows the pooled rates of cannabis-associated psychotic symptoms (CAPS) across the three different study designs. Estimates displayed in purple are the pooled rates (in %, 95% Confidence Interval) for each symptom profile of CAPS (psychosis-like symptoms, hallucinations, paranoia) separately. Estimates displayed in blue are the results from models pooling together all estimates on rates of CAPS (including psychosis-like symptoms, paranoia, hallucinations and delusions) per study design. k=number of published studies included in the model; n=number of included (non-overlapping) individuals.

sFigure 3. Peters' test for publication bias (rates of CAPS)

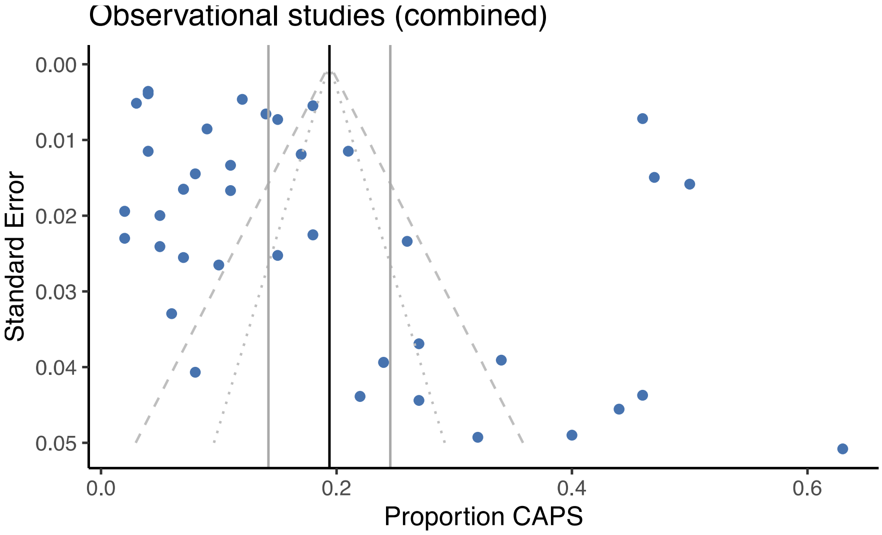

Test for publication bias: p=0.83
Pooled rate (unadjusted): d=19.39% (95%CI 14.22; 24.57)

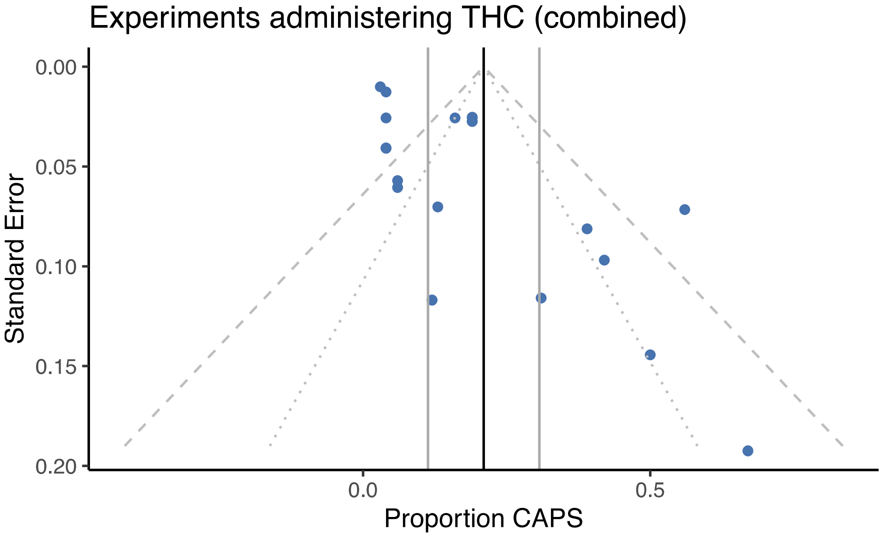

Test for publication bias: p=0.33
Pooled rate (unadjusted): d=20.99% (95%CI 11.29; 30.7)

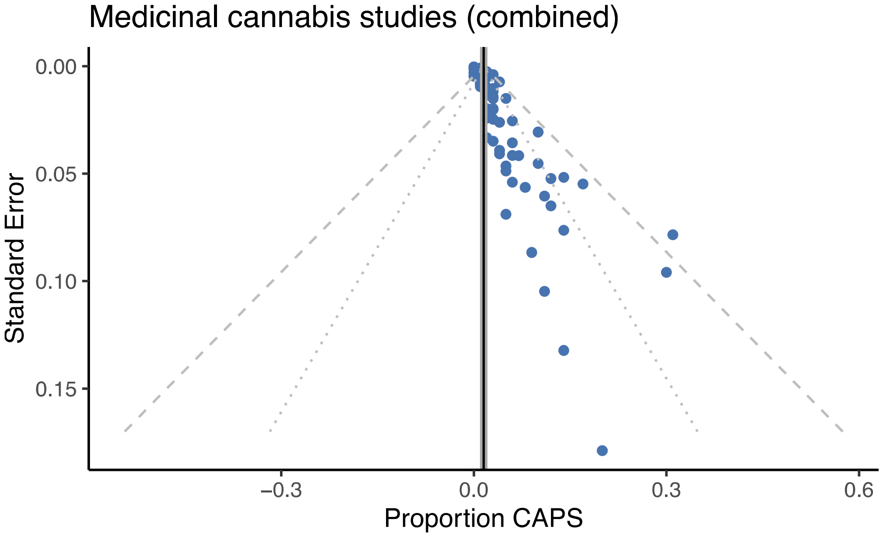

Test for publication bias: p=0.0011
Pooled rate (unadjusted): d=1.53% (95%CI 1.15; 1.9)
Trim−and−fill corrected pooled rate of CAPS: d=1.18% (95% CI 0.83; 1.54)

Funnel plots show each meta-analytical model on rates of CAPS separately. The individual proportions (x-axis) of CAPS are plotted against the standard error (y-axis). The black and grey horizontal lines indicate the pooled proportion of CAPS and their corresponding 95% confidence interval. The dashed and dotted lines highlight the 95% and 99% confidence interval region, respectively. Whenever risk of publication bias was indicated (Peters' test for publication bias *p*<0.05), the trim-and-fill method was applied. The trim-and-fill adjusted pooled rate of CAPS is included in the caption of the funnel plot. *p*-values for publication bias are two-sided and uncorrected for multiple testing.

sFigure 4. Egger's test for publication bias (predictors of CAPS)

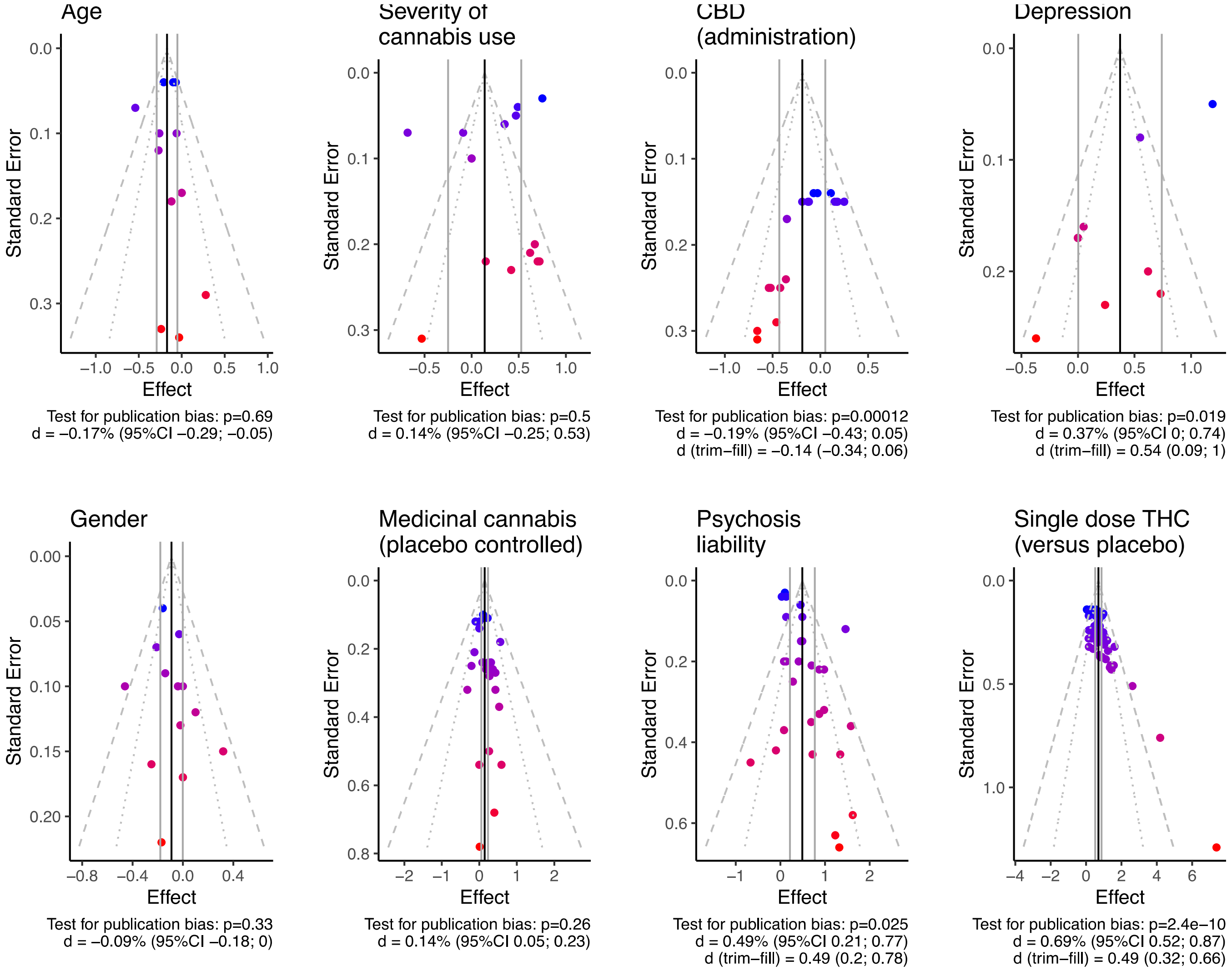

Egger's plot for publications bias, including the individual estimates of Cohen's *d* (x-axis) and the corresponding standard errors (y-axis). *p*-values for publication bias are two-sided and uncorrected for multiple testing.

sFigure 5. Leave-one-out and outlier analysis

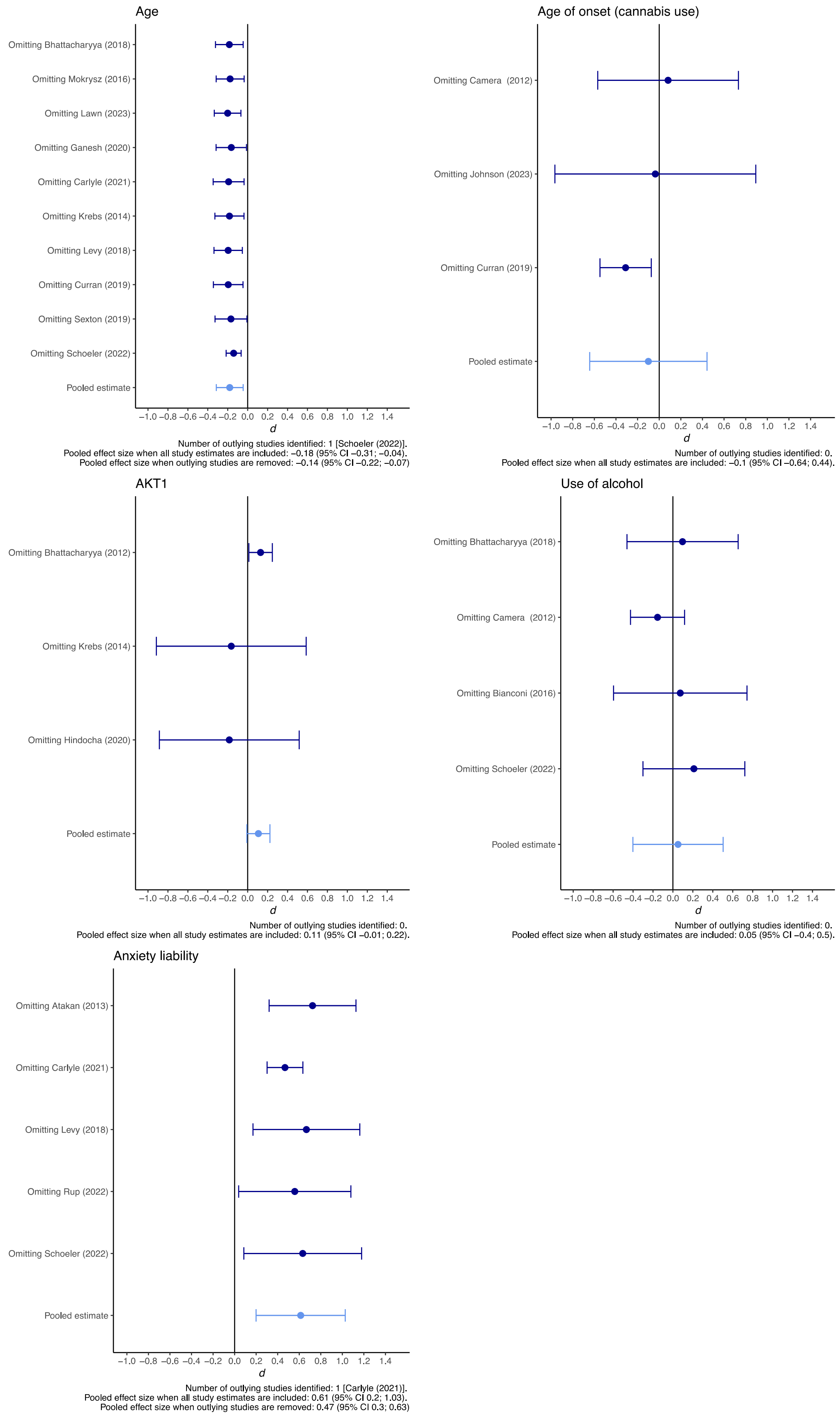

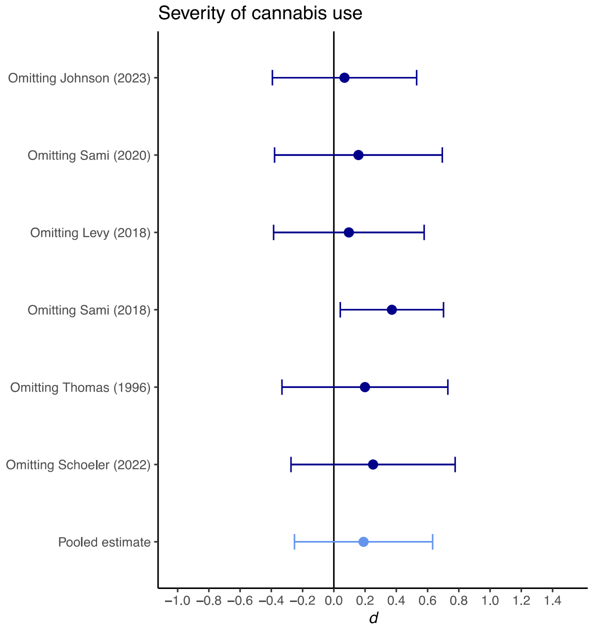

Number of outlying studies identified: 2 [Johnson (2023), Sami (2018)].
Pooled effect size when all study estimates are included: 0.19 (95% CI −0.25; 0.63).
Pooled effect size when outlying studies are removed: 0.25 (95% CI −0.07; 0.58)

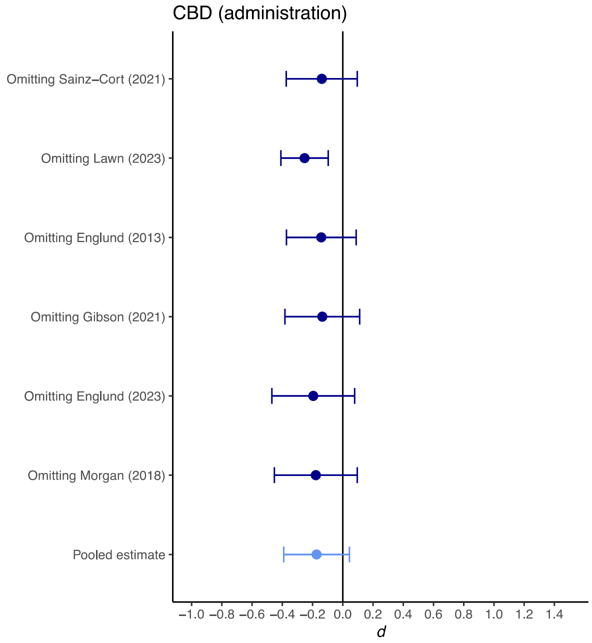

Number of outlying studies identified: 0.
Pooled effect size when all study estimates are included: −0.17 (95% CI −0.39; 0.04).

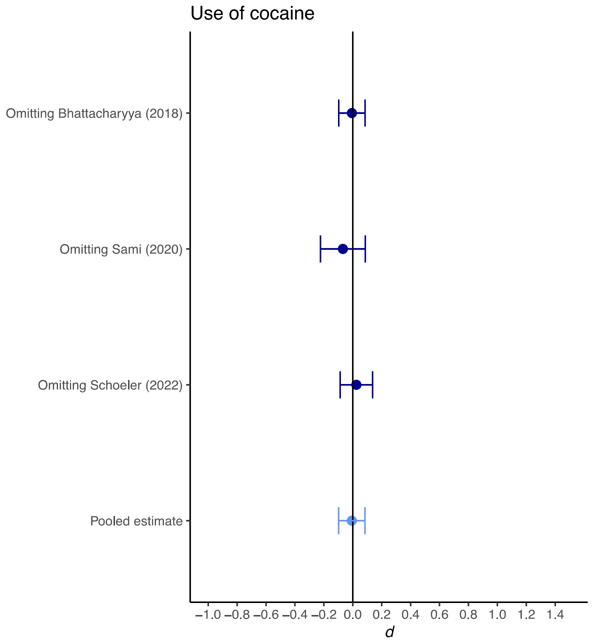

Number of outlying studies identified: 0.
Pooled effect size when all study estimates are included: −0.01 (95% CI −0.1; 0.08).

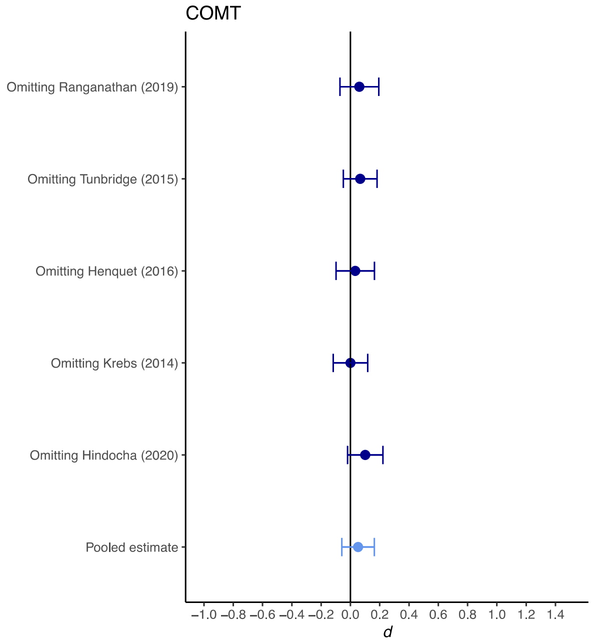

Number of outlying studies identified: 0.
Pooled effect size when all study estimates are included: 0.05 (95% CI −0.06; 0.16).

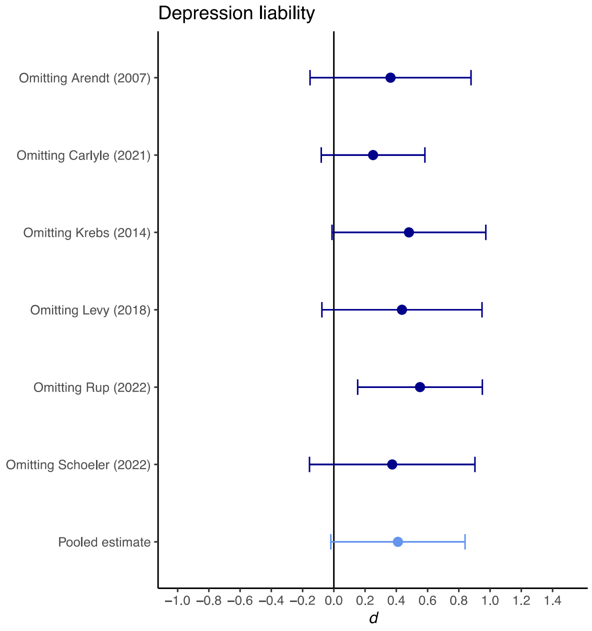

Number of outlying studies identified: 1 [Carlyle (2021)].
Pooled effect size when all study estimates are included: 0.41 (95% CI −0.02; 0.84).
Pooled effect size when outlying studies are removed: 0.25 (95% CI −0.08; 0.58)

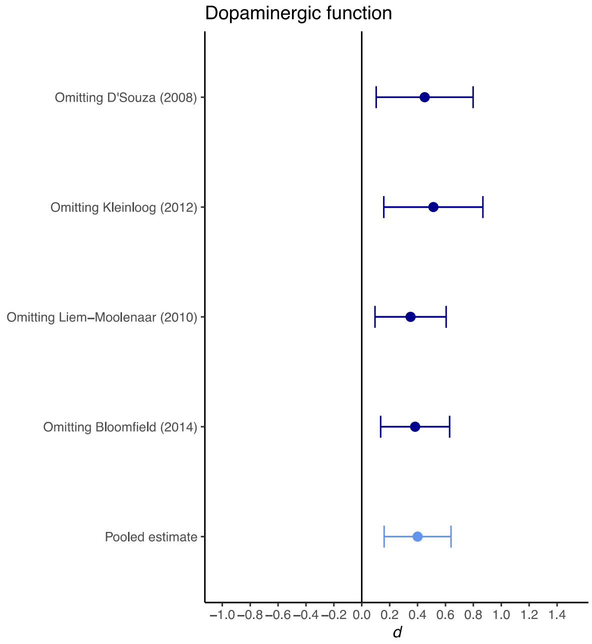

Number of outlying studies identified: 0.
Pooled effect size when all study estimates are included: 0.4 (95% CI 0.16; 0.64).

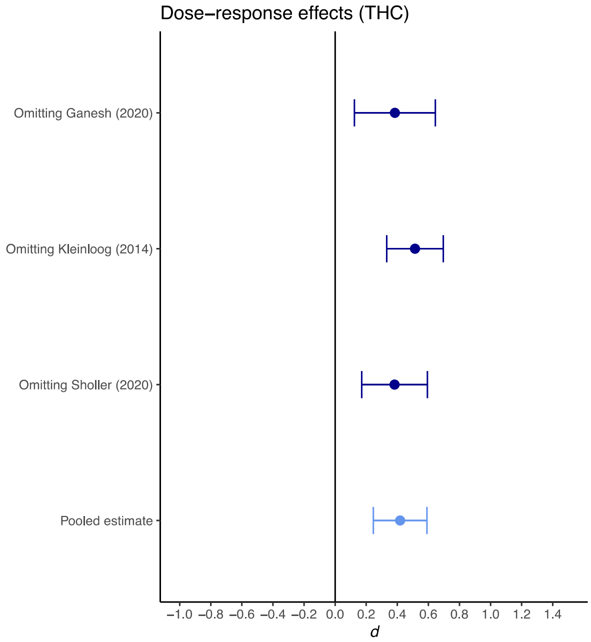

Number of outlying studies identified: 0.
Pooled effect size when all study estimates are included: 0.42 (95% CI 0.25; 0.59).

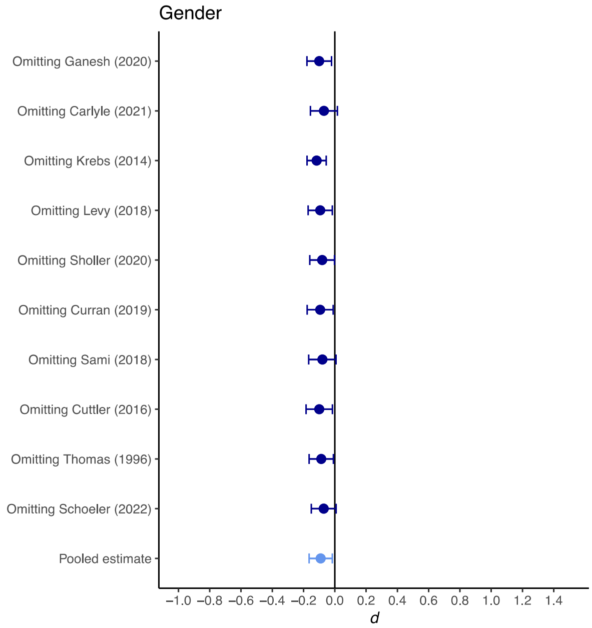

Number of outlying studies identified: 1 [Krebs (2014)].
Pooled effect size when all study estimates are included: −0.09 (95% CI −0.16; −0.02).
Pooled effect size when outlying studies are removed: −0.12 (95% CI −0.18; −0.06)

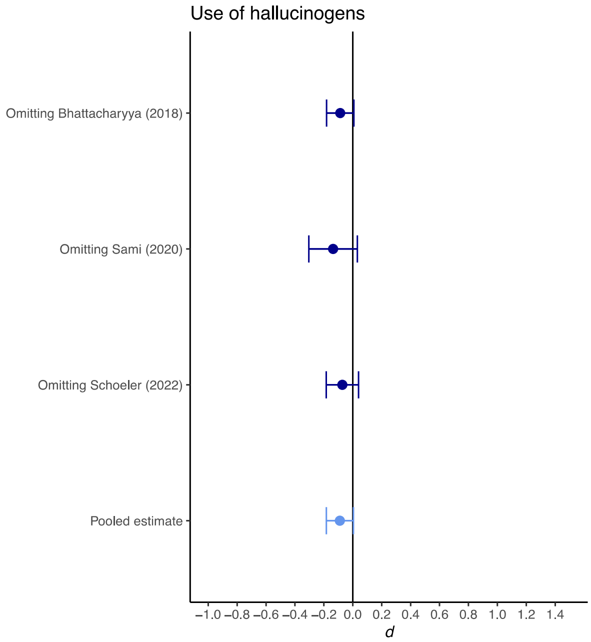

Number of outlying studies identified: 0.
Pooled effect size when all study estimates are included: −0.09 (95% CI −0.18; 0).

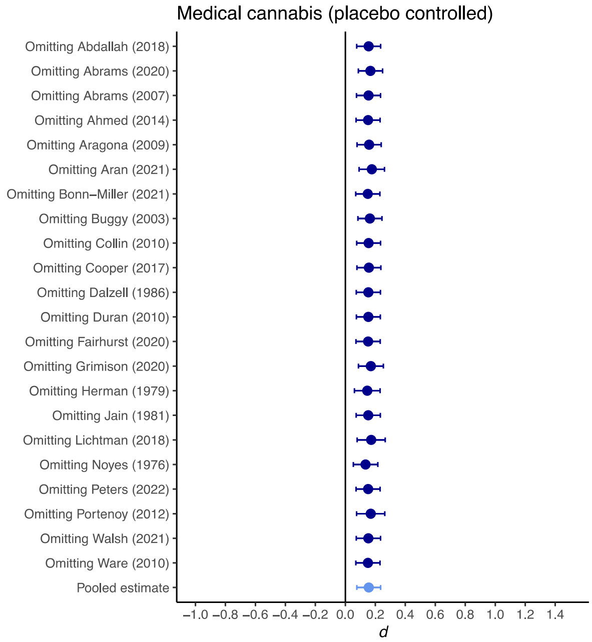

Number of outlying studies identified: 0.
Pooled effect size when all study estimates are included: 0.16 (95% CI 0.08; 0.24).

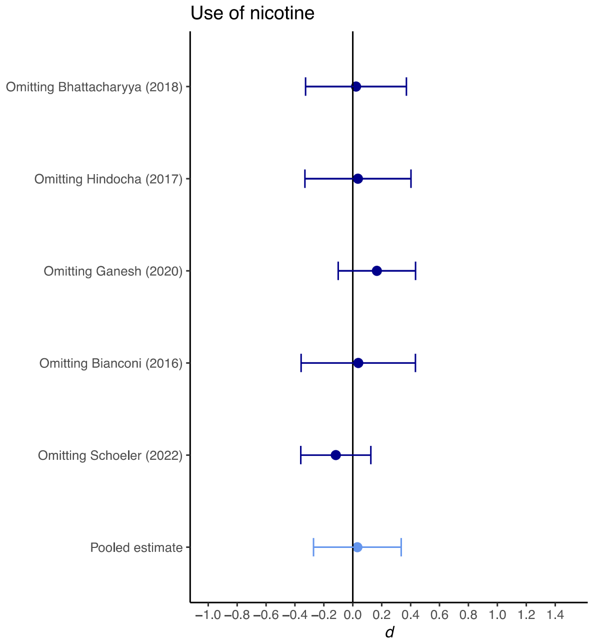

Number of outlying studies identified: 0.
Pooled effect size when all study estimates are included: 0.03 (95% CI −0.27; 0.33).

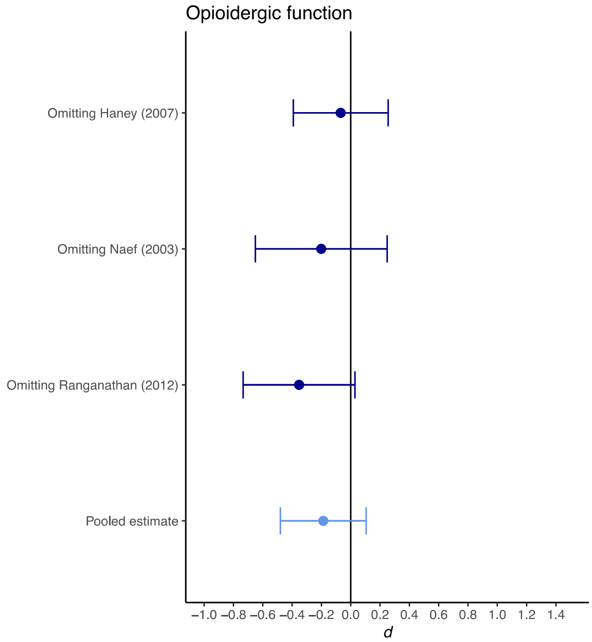

Number of outlying studies identified: 0.
Pooled effect size when all study estimates are included: −0.19 (95% CI −0.48; 0.11).

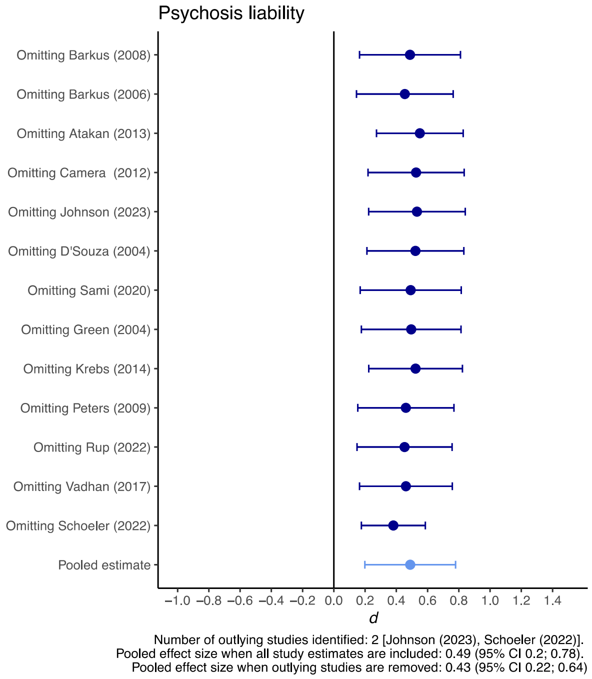

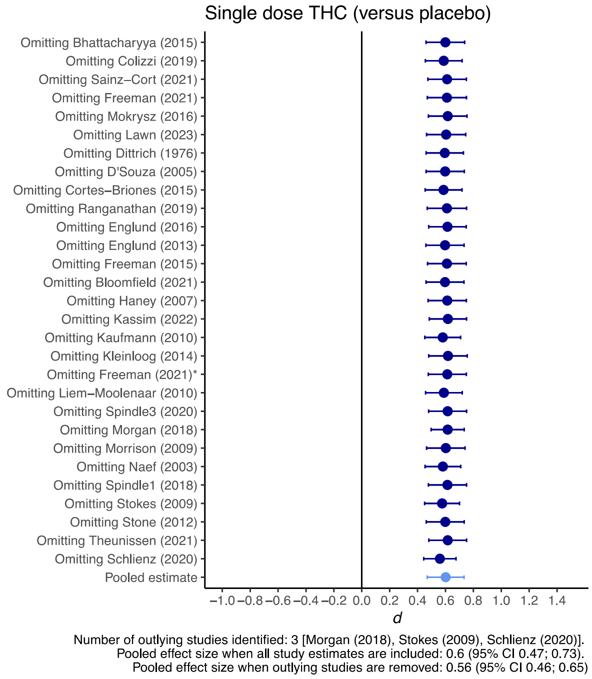

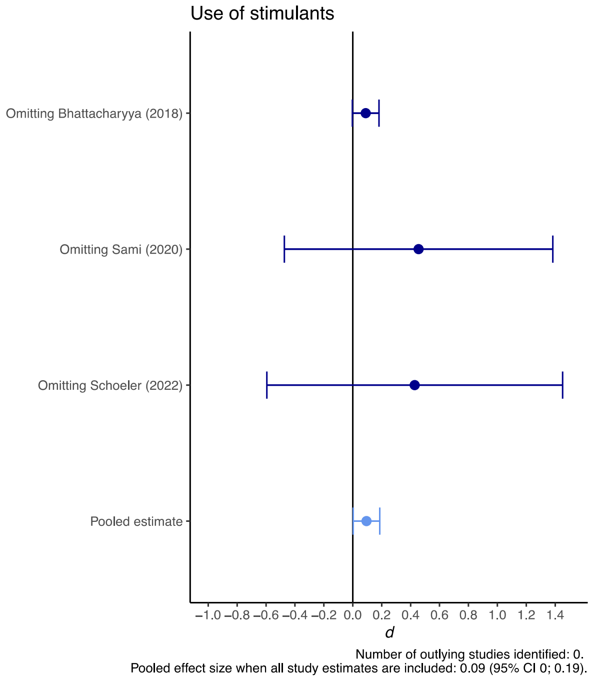

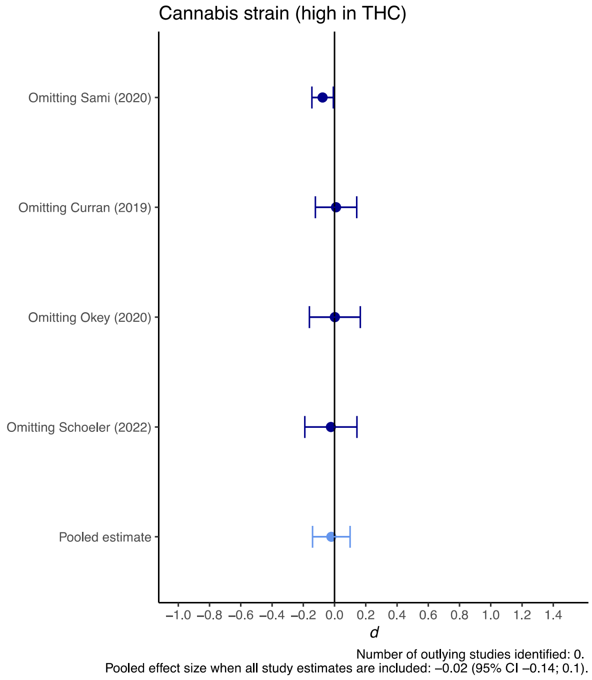

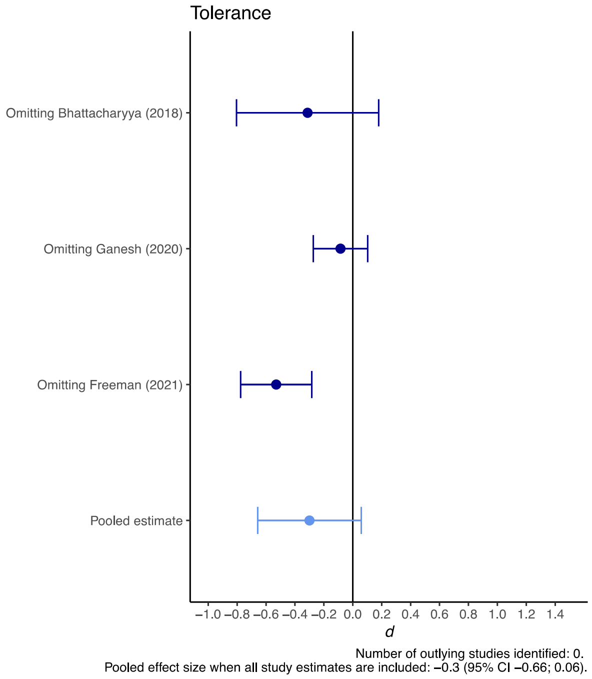

Note. Pooled estimates of Cohen's *d* (x-axis, with corresponding 95% Confidence Intervals) obtained from leave-one-out analysis. Information on sample sizes and study characteristics can be found in sTable 4.

sFigure 6. Comparison of estimates of Cohen's d obtained for predictors of CAPS, with varying levels of withing-subject correlations

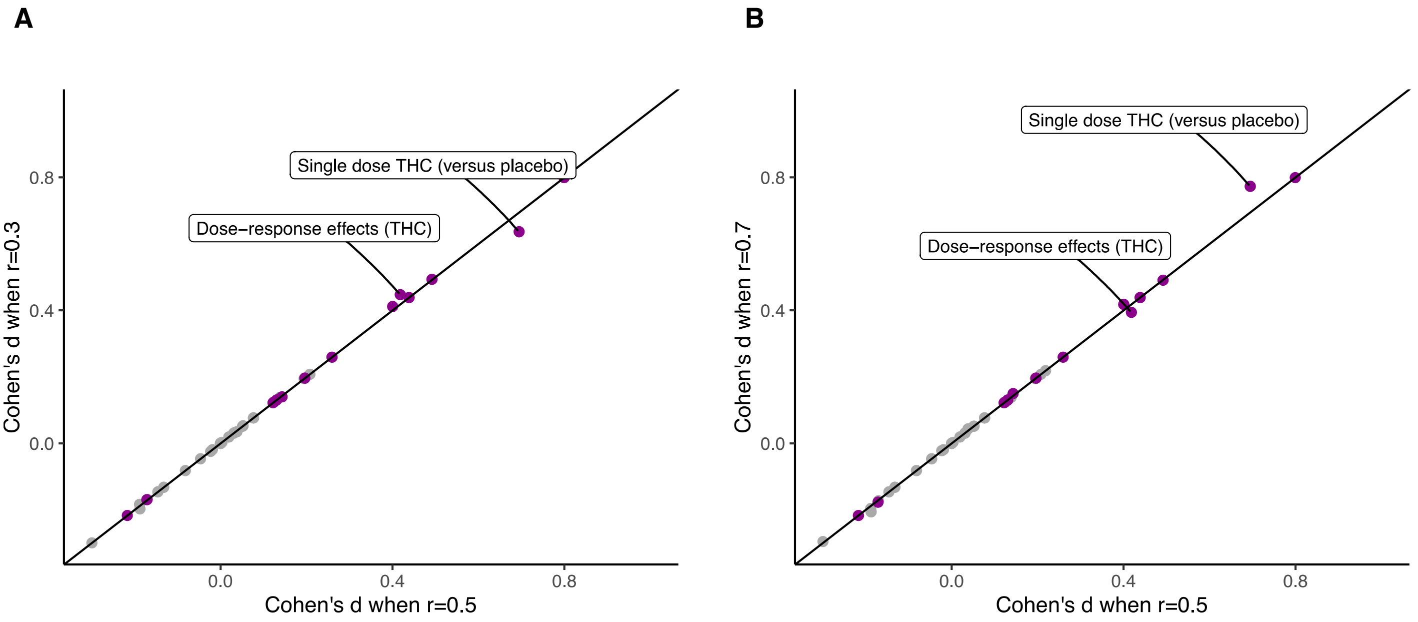

Highlighted in magenta are estimates of Cohen's *d* that are significant in both analyses, including the main analysis (where *r*=0.5) and the corresponding sensitivity analysis (with either *r*=0.3 as shown in **Panel A** or *r*=0.7 as shown in **Panel B**). Points that are highlighted with a label index those effect estimates where $|d_{MAIN} - d_{SENSITIVITY}|>0.02$.

sFigure 7. Triangulation of findings across different study designs

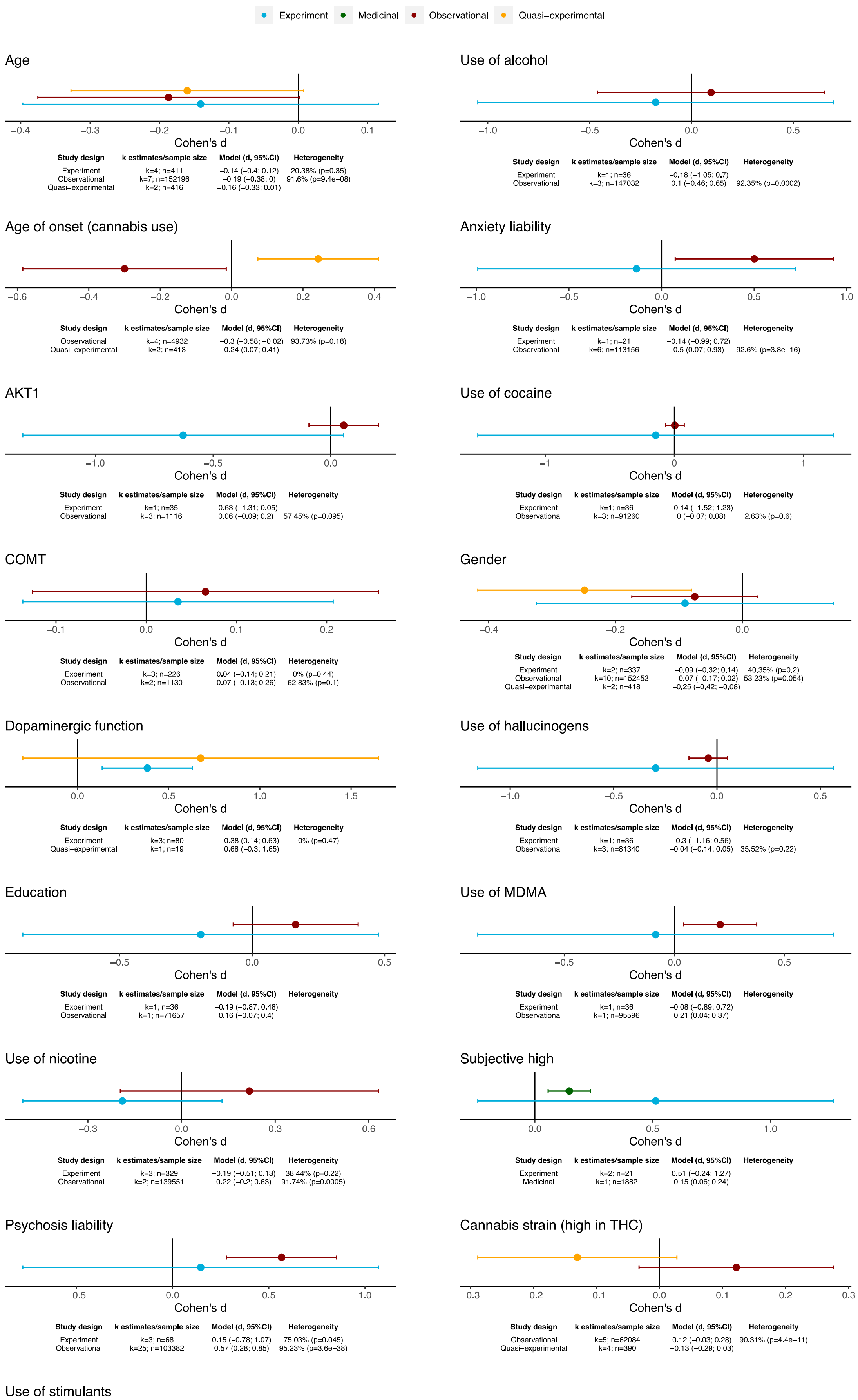

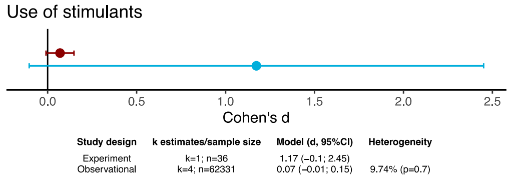

Note. Shown are the pooled estimates of Cohen's *d* when estimated separately for each of the different study designs. I^2 statistic (scale of 0 to 100) indexes the level of heterogeneity across the estimates included in the meta-analysis. k=number of study estimates included in the model; n=number of included (non-overlapping) individuals. Information on study characteristics can be found in sTable 4. n=sample size included per meta-analytical model. *p*-values for heterogeneity are two-sided and uncorrected for multiple testing.

Questionnaires used to assess cannabis-associated psychotic symptoms

SDES	Subjective Drug Effect Scales[24]
ARS	Adverse Reactions Scale[25]
SCL-90-R	Symptom Checklist-90 Revised (psychosis)[26]
PSI	Psychotomimetic States Inventory[27]
BPRS	Brief Psychiatric Rating Scale[28]
CAPE	Community Assessment of Psychic Experiences[29]
CEQ	Cannabis Experiences Questionnaire[30]
CAPE	Community Assessment of Psychic Experiences-state
ASC	Altered States of Consciousness[31]
VAS	Visual Analogue Scale[32,33]
TEAEs	Treatment Emergent Adverse Events[34]
DEQ	Drug Effect Questionnaire[35]
SECQ	Subjective Effects of Cannabis Questionnaire[36]
CSE	Cannabis Subjective Experience Questionnaire[13]
SSPS	Social Situation Paranoia Scale[37]
SDES	Subjective Drug Effects Questionnaire[38]
PANSS	Positive and negative syndrome scale for schizophrenia[39]
LSHS-R	Launay-Slade Hallucination Scale-Revised[40]
MIS	Magical Ideation Scale[41]
PAS	Perceptual Aberrations Scale[42]
POMS	Profile of Mood States questionnaire[43]
CanTox-17	Cannabis intoxication effects[44]
SSAGA	Semi-Structured Assessment for the Genetics of Alcoholism[45]

sReferences

1. Borenstein M, Hedges L V, Higgins JPT, Rothstein HR. *Introduction to Meta-Analysis*. John Wiley & Sons; 2011.
2. Jacobs P, Viechtbauer W. Estimation of the biserial correlation and its sampling variance for use in meta-analysis. *Res Synth Methods*. 2017;8(2):161-180. doi:10.1002/jrsm.1218
3. Goulet-Pelletier J-C, Cousineau D. A review of effect sizes and their confidence intervals, Part I: The Cohen's d family. *Quant Methods Psychol*. 2018;14(4):242-265. doi:10.20982/tqmp.14.4.p242
4. Rutherford A. Designing experiments and analyzing data: A model comparison perspective. *Br J Math Stat Psychol*. 2004;57:185.
5. Borenstein M, Hedges LV, Higgins JPT, Rothstein HR. Multiple Outcomes or Time-Points within a Study. In: *Introduction to Meta-Analysis*. John Wiley & Sons, Ltd; :225-238. doi:10.1002/9780470743386.ch24
6. Bianconi F, Bonomo M, Marconi A, et al. Differences in cannabis-related experiences between patients with a first episode of psychosis and controls. *Psychol Med*. 2016;46(5):995-1003. doi:10.1017/S0033291715002494
7. D'Souza DC, Perry E, MacDougall L, et al. The psychotomimetic effects of intravenous delta-9-tetrahydrocannabinol in healthy individuals: Implications for psychosis. *Neuropsychopharmacology*. 2004;29(8):1558-1572. doi:10.1038/sj.npp.1300496
8. Schoeler T, Ferris J, Winstock AR. Rates and correlates of cannabis-associated psychotic symptoms in over 230,000 people who use cannabis. *Transl Psychiatry*. 2022;12(1):369. doi:10.1038/s41398-022-02112-8
9. Rup J, Freeman TP, Perlman C, Hammond D. Cannabis and mental Health: Adverse outcomes and self-reported impact of cannabis use by mental health status. *Subst Use Misuse*. 2022;57(5):719-729. doi:10.1080/10826084.2022.2034872
10. Arendt M, Rosenberg R, Fjordback L, et al. Testing the self-medication hypothesis of depression and aggression in cannabis-dependent subjects. *Psychol Med*. 2007;37(7):935-945. doi:10.1017/S0033291706009688
11. Vadhan NP, Corcoran CM, Bedi G, Keilp JG, Haney M. Acute effects of smoked marijuana in marijuana smokers at clinical high-risk for psychosis: A preliminary study. *Psychiatry Res*. 2017;257:372-374. doi:10.1016/j.psychres.2017.07.070
12. Peters BD, de Koning P, Dingemans P, Becker H, Linszen DH, de Haan L. Subjective effects of cannabis before the first psychotic episode. *Aust New Zeal J Psychiatry*. 2009;43(12):1155-1162. doi:10.3109/00048670903179095
13. Krebs MO, Morvan Y, Jay T, Gaillard R, Kebir O. Psychotomimetic effects at initiation of cannabis use are associated with cannabinoid receptor 1 (CNR1) variants in healthy students. *Mol Psychiatry*. 2014;19(4):402-403. doi:10.1038/mp.2013.188
14. Carlyle M, Constable T, Walter ZC, Wilson J, Newland G, Hides L. Cannabis-induced dysphoria/paranoia mediates the link between childhood trauma and psychotic-like experiences in young cannabis users. *Schizophr Res*. 2021;238:178-184. doi:10.1016/j.schres.2021.10.011
15. Atakan Z, Bhattacharyya S, Allen P, et al. Cannabis affects people differently: Inter-subject variation in the psychotogenic effects of DELTA9-tetrahydrocannabinol: A functional magnetic resonance imaging study with healthy volunteers. Arseneault Battisti, Bhattacharyya, Bhattacharyya, Bhattacharyya, Bhattacharyya, Borgwardt, Brammer, Bullmore, Cascini, Caspi, Chambers, Chesher, Curran, D'Souza, D'Souza, Degenhardt, Enticott, Fleck, Fusar-Poli, Fusar-Poli, Fusar-Poli, Gray, Green, Grot B, ed. *Psychol Med*. 2013;43(6):1255-1267. doi:http://dx.doi.org/10.1017/S0033291712001924
16. Camera AA, Tomaselli V, Fleming J, et al. Correlates to the variable effects of cannabis in young adults: a preliminary study. *Harm Reduct J*. 2012;9(1):15. doi:10.1186/1477-7517-9-15
17. Johnson EC, Colbert SMC, Jeffries PW, et al. Associations between cannabis use, polygenic liability for schizophrenia, and cannabis-related experiences in a sample of cannabis users. *Schizophr Bull*. 2023;49(3):778-787. doi:10.1093/schbul/sbac196

18. Trubetskoy V, Pardiñas AF, Qi T, et al. Mapping genomic loci implicates genes and synaptic biology in schizophrenia. *Nature*. 2022;604(7906):502-508. doi:10.1038/s41586-022-04434-5
19. Viechtbauer W. Conducting Meta-Analyses in R with the metafor Package. *J Stat Softw*. 2010;36(3). doi:10.18637/jss.v036.i03
20. Peters JL. Comparison of two methods to detect publication bias in meta-analysis. *JAMA*. 2006;295(6):676. doi:10.1001/jama.295.6.676
21. Tang J-L, Liu JL. Misleading funnel plot for detection of bias in meta-analysis. *J Clin Epidemiol*. 2000;53(5):477-484. doi:10.1016/S0895-4356(99)00204-8
22. Duval S, Tweedie R. Trim and Fill: A simple funnel-plot-based method of testing and adjusting for publication bias in meta-analysis. *Biometrics*. 2000;56(2):455-463. doi:10.1111/j.0006-341X.2000.00455.x
23. Harrer M, Cuijpers P, Furukawa T, Ebert DD. Dmetar: Companion R package for the guide'doing meta-analysis in R'. *R Packag version 00*. 2019;9000.
24. Waskow IE. Psychological effects of Tetrahydrocannabinol. *Arch Gen Psychiatry*. 1970;22(2):97. doi:10.1001/archpsyc.1970.01740260001001
25. LaFrance EM, Stueber A, Glodosky NC, Mauzay D, Cuttler C. Overbaked: assessing and predicting acute adverse reactions to Cannabis. *J Cannabis Res*. 2020;2(1):3. doi:10.1186/s42238-019-0013-x
26. Derogatis LR, Unger R. Symptom Checklist-90-Revised. In: *The Corsini Encyclopedia of Psychology*. Wiley; 2010:1-2. doi:10.1002/9780470479216.corpsy0970
27. Mason OJ, Morgan CJM, Stefanovic A, Curran HV. The Psychotomimetic States Inventory (PSI): Measuring psychotic-type experiences from ketamine and cannabis. *Schizophr Res*. 2008;103(1-3):138-142. doi:10.1016/j.schres.2008.02.020
28. Overall JE, Gorham DR. The Brief Psychiatric Rating Scale. *Psychol Rep*. 1962;10(3):799-812. doi:10.2466/pr0.1962.10.3.799
29. Konings M, Bak M, Hanssen M, Van Os J, Krabbendam L. Validity and reliability of the CAPE: a self-report instrument for the measurement of psychotic experiences in the general population. *Acta Psychiatr Scand*. 2006;114(1):55-61. doi:10.1111/j.1600-0447.2005.00741.x
30. Quinn CA, Wilson H, Cockshaw W, Barkus E, Hides L. Development and validation of the cannabis experiences questionnaire – Intoxication effects checklist (CEQ-I) short form. *Schizophr Res*. 2017;189:91-96. doi:10.1016/j.schres.2017.01.048
31. Dittrich A. The Standardized Psychometric Assessment of Altered States of Consciousness (ASCs) in humans. *Pharmacopsychiatry*. 1998;31(S 2):80-84. doi:10.1055/s-2007-979351
32. Bowdle AT, Radant AD, Cowley DS, Kharasch ED, Strassman RJ, Roy-Byrne PP. Psychedelic effects of Ketamine in healthy volunteers. *Anesthesiology*. 1998;88(1):82-88. doi:10.1097/00000542-199801000-00015
33. Norris H. The action of sedatives on brain stem oculomotor systems in man. *Neuropharmacology*. 1971;10(2):181-191. doi:10.1016/0028-3908(71)90039-6
34. van Amerongen G, Siebenga P, de Kam ML, Hay JL, Groeneveld GJ. Effect profile of paracetamol, Δ9-THC and promethazine using an evoked pain test battery in healthy subjects. *Eur J Pain*. 2018;22(7):1331-1342. doi:10.1002/ejp.1222
35. Vandrey R, Herrmann ES, Mitchell JM, et al. Pharmacokinetic profile of oral cannabis in humans: Blood and oral fluid disposition and relation to pharmacodynamic outcomes. *J Anal Toxicol*. 2017;41(2):83-99. doi:10.1093/jat/bkx012
36. Stone JM, Morrison PD, Brugger S, et al. Communication breakdown: delta-9 tetrahydrocannabinol effects on pre-speech neural coherence. *Mol Psychiatry*. 2012;17(6):568-569. doi:10.1038/mp.2011.141
37. Freeman D, Pugh K, Green C, Valmaggia L, Dunn G, Garety P. A measure of state persecutory ideation for experimental studies. *J Nerv Ment Dis*. 2007;195(9):781-784. doi:10.1097/NMD.0b013e318145a0a9
38. Noyes R, Brunk SF, Avery DH, Canter A. Psychologic effects of oral delta-9-tetrahydrocannabinol in advanced cancer patients. *Compr Psychiatry*. 1976;17(5):641-646. doi:10.1016/S0010-440X(76)80008-9

39. Kay SR, Fiszbein A, Opler LA. The Positive and Negative Syndrome Scale (PANSS) for Schizophrenia. *Schizophr Bull*. 1987;13(2):261-276. doi:10.1093/schbul/13.2.261
40. Morrison AP, Wells A, Nothard S. Launay-Slade Hallucination Scale-Revised. *Br J Clin Psychol*.
41. Eckblad M, Chapman LJ. Magical ideation as an indicator of schizotypy. *J Consult Clin Psychol*. 1983;51(2):215-225. doi:10.1037/0022-006X.51.2.215
42. Chapman LJ, Chapman JP, Raulin ML. Perceptual aberration scale. *Unpubl manuscript, Univ Wisconsin—Madison*. Published online 1978.
43. Douglas MM. Manual for the profile of mood states. *Educ Ind Test Serv*. 1971;1984.
44. Schüler M, Moritz S, Schnell T. Development and initial validation of a reliable German self-report measure to assess acute cannabis intoxication-effects (CanTox-17). *Int J Methods Psychiatr Res*. 2022;31(3). doi:10.1002/mpr.1925
45. Bucholz KK, Cadoret R, Cloninger CR, et al. Semi-Structured Assessment for the Genetics of Alcoholism. *J Stud Alcohol*.
