# Supplementary material for: Assessing rates and predictors of cannabis-associated psychotic symptoms across observational, experimental and medical research
Source: Nat Ment Health. 2024 Jun 3;2(7):865–76. doi: 10.1038/s44220-024-00261-x (PMC11236708; doi:10.1038/s44220-024-00261-x)
Supplement: Supplementary file 2 — Reporting Summary [file 44220_2024_261_MOESM2_ESM.pdf]

nature portfolio

Corresponding author(s): Tabea Schoeler

Last updated by author(s): Mar 5, 2024

Reporting Summary

Nature Portfolio wishes to improve the reproducibility of the work that we publish. This form provides structure for consistency and transparency in reporting. For further information on Nature Portfolio policies, see our Editorial Policies and the Editorial Policy Checklist.

Statistics

For all statistical analyses, confirm that the following items are present in the figure legend, table legend, main text, or Methods section.

n/a	Confirmed	
☐	☒	The exact sample size (*n*) for each experimental group/condition, given as a discrete number and unit of measurement
☐	☒	A statement on whether measurements were taken from distinct samples or whether the same sample was measured repeatedly
☐	☒	The statistical test(s) used AND whether they are one- or two-sided<br>*Only common tests should be described solely by name; describe more complex techniques in the Methods section.*
☒	☐	A description of all covariates tested
☒	☐	A description of any assumptions or corrections, such as tests of normality and adjustment for multiple comparisons
☐	☒	A full description of the statistical parameters including central tendency (e.g. means) or other basic estimates (e.g. regression coefficient) AND variation (e.g. standard deviation) or associated estimates of uncertainty (e.g. confidence intervals)
☐	☒	For null hypothesis testing, the test statistic (e.g. *F*, *t*, *r*) with confidence intervals, effect sizes, degrees of freedom and *P* value noted<br>*Give P values as exact values whenever suitable.*
☒	☐	For Bayesian analysis, information on the choice of priors and Markov chain Monte Carlo settings
☐	☒	For hierarchical and complex designs, identification of the appropriate level for tests and full reporting of outcomes
☐	☒	Estimates of effect sizes (e.g. Cohen's *d*, Pearson's *r*), indicating how they were calculated

Our web collection on statistics for biologists contains articles on many of the points above.

Software and code

Policy information about availability of computer code

Data collection	Data was extracted from published studies, including experiments testing the effect of THC, observational studies assessing recreational cannabis users and studies monitoring adverse events in individuals using medicinal cannabis products.
Data analysis	All customized functions used to convert effect estimates to a common effect size (Cohen's d) are available on the study repository (https://github.com/TabeaSchoeler/TS2023_MetaCAPS). Data analysis was performed in R (version 4.3.1), using meta-analytical models to synthesize the extracted data. Fitted were random-effects models (REM) or multilevel REM models (MREM) as implemented in the R package metafor (version 4.4.0). We derived rates of cannabis-associated psychotic symptoms and the corresponding confidence intervals using the function BinomCI() and the Clopper-Pearson method as implemented in the R-package DescTools (version 0.99.52).

For manuscripts utilizing custom algorithms or software that are central to the research but not yet described in published literature, software must be made available to editors and reviewers. We strongly encourage code deposition in a community repository (e.g. GitHub). See the Nature Portfolio guidelines for submitting code & software for further information.

Data

Policy information about availability of data

All manuscripts must include a data availability statement. This statement should provide the following information, where applicable:

- Accession codes, unique identifiers, or web links for publicly available datasets
- A description of any restrictions on data availability
- For clinical datasets or third party data, please ensure that the statement adheres to our policy

All data and analytical scripts associated with the current submission is accessible via the study repository (https://github.com/TabeaSchoeler/TS2023_MetaCAPS)

Research involving human participants, their data, or biological material

Policy information about studies with human participants or human data. See also policy information about sex, gender (identity/presentation), and sexual orientation and race, ethnicity and racism.

Reporting on sex and gender	NA
Reporting on race, ethnicity, or other socially relevant groupings	NA
Population characteristics	NA
Recruitment	NA
Ethics oversight	NA

Note that full information on the approval of the study protocol must also be provided in the manuscript.

Field-specific reporting

Please select the one below that is the best fit for your research. If you are not sure, read the appropriate sections before making your selection.

☒ Life sciences ☐ Behavioural & social sciences ☐ Ecological, evolutionary & environmental sciences

For a reference copy of the document with all sections, see nature.com/documents/nr-reporting-summary-flat.pdf

Life sciences study design

All studies must disclose on these points even when the disclosure is negative.

Sample size	163 studies (including 210,287 independent participants) were included, comprising cannabis-exposed individuals taking part in either observational (n=174,300), experimental research (n=2,009) or medicinal cannabis (n=33,502) research. Independent study participants are defined as the maximum number of participants available for an underlying study sample assessed in one or more of the included studies.
Data exclusions	We excluded studies that did not provide results to estimate rates of CAPS (i.e., the proportion of cannabis-exposed individuals reporting CAPS) or effect sizes (Cohen's d) for factors predicting cannabis-associated psychotic symptoms (CAPS). CAPS was defined as the occurrence of hallucinations, paranoia and/or delusions during cannabis intoxication.
Replication	This work synthesized the existing evidence on rates and predictors of CAPS. The level of replicability (i.e., heterogeneity) across the studies was assessed using the heterogeneity index I2.
Randomization	NA - this study summarizes existing findings and did not include experimental randomization
Blinding	NA - this study summarizes existing findings and did not include experimental randomization

Reporting for specific materials, systems and methods

We require information from authors about some types of materials, experimental systems and methods used in many studies. Here, indicate whether each material, system or method listed is relevant to your study. If you are not sure if a list item applies to your research, read the appropriate section before selecting a response.

Materials & experimental systems

n/a	Involved in the study
☒	☐ Antibodies
☒	☐ Eukaryotic cell lines
☒	☐ Palaeontology and archaeology
☒	☐ Animals and other organisms
☒	☐ Clinical data
☒	☐ Dual use research of concern
☒	☐ Plants

Methods

n/a	Involved in the study
☒	☐ ChIP-seq
☒	☐ Flow cytometry
☒	☐ MRI-based neuroimaging
